# Supporting the IOC Consensus Statement on Mental Health in Elite Athletes: A Systematic Review and Meta-Analysis on the Prevalence of Mental Health Symptoms in Elite Sports

**DOI:** 10.3390/sports14070296

**Published:** 2026-07-10

**Authors:** Vincent Gouttebarge, Sharaisha C. Bilgoe, Paul Gorczynski, Mary E. Hitchcock, Margot Putukian, Claudia L. Reardon, Gino Kerkhoffs

**Affiliations:** 1 Amsterdam UMC Location University of Amsterdam, Department of Orthopedic Surgery and Sports Medicine, 1105AZ Amsterdam, The Netherlands; 2 Section Sports Medicine, University of Pretoria, Pretoria 0028, South Africa; 3 FIFPRO (Football Players Worldwide), 2132LR Hoofddorp, The Netherlands; 4 Amsterdam Collaboration for Health & Safety in Sports (ACHSS), IOC Research Center, 1105AZ Amsterdam, The Netherlands; 5 School of Human Sciences, University of Greenwich, London SE10 9LS, UK; 6 Ebling Library, University of Wisconsin School of Medicine and Public Health, Madison, WI 53705-2221, USA; 7 US Soccer Federation, Princeton University, Princeton, NJ 08544, USA; mputukian@gmail.com; 8 Department of Psychiatry, University of Wisconsin School of Medicine and Public Health, Madison, WI 53705-2221, USA; clreardon@wisc.edu; 9 Academic Center for Evidence Based Sports Medicine (ACES), 1105AZ Amsterdam, The Netherlands

**Keywords:** mental health symptoms and disorders, sports, elite athletes, prevalence, meta-analysis

## Abstract

We explored the epidemiological evidence on the prevalence of mental health symptoms among current and former elite athletes as well as among their entourage members. Methods: A systematic review and meta-analysis were conducted in PubMed, PsycINFO, SportDiscus and Scopus to retrieve original quantitative studies that (i) were written in English, (ii) were conducted exclusively among current or former elite athletes and/or their entourage members, and (iii) presented prevalence rates of mental health symptoms. Results: In total, 72 studies were included, focusing on self-reported mental health symptoms (not on clinically diagnosed mental health disorders). Meta-analyses comprising 2596 to 10,927 current elite athletes showed that the prevalence of mental health symptoms ranged from 4% for drug misuse to 33% for distress. Meta-analyses comprising 2070 to 3405 former elite athletes showed that the prevalence of mental health symptoms ranged from 12% for depression to 28% for alcohol misuse. Because of the considerable heterogeneity among the included studies, the pooled prevalence estimates from these meta-analyses should be interpreted cautiously, as they summarize highly heterogeneous populations. Mental health symptoms were found to also be common among entourage members, with prevalence rates ranging from 5% for depression or anxiety to 53% for alcohol misuse in high-performance staff (including coaches) and reaching up to 36–58% for burnout in healthcare professionals working during the Paralympic games. Conclusions: Showing considerable heterogeneity across included studies, our systematic review and meta-analysis established that mental health symptoms are commonly reported by current and former elite athletes, as well as by their high-performance staff (including coaches) and medical staff. This warrants the implementation of various tailored resources, such as mental health literacy, screening programs and the availability of interdisciplinary medical and psychological support.

## 1. Introduction

Elite athletes, including those competing at the collegiate, Olympic, and professional levels, encounter multiple sport-specific and general stressors throughout their careers. These stressors may contribute to the development of mental health symptoms or disorders both during active participation in sport and following retirement from competition [1,2]. Mental health symptoms refer to self-reported adverse thoughts, emotions, and/or behaviors that do not fulfill established diagnostic criteria for a mental disorder and may not result in clinically significant distress or impairment in daily functioning [2,3]. In the scientific literature, nearly all studies conducted in current and former elite athletes refer to self-reported mental health symptoms [2]. In 2019, a systematic review and meta-analysis was conducted to support the first IOC consensus statement on mental health in elite athletes [1]. Based on a limited number of epidemiological studies, the prevalence rates of mental health symptoms ranged from 19% for alcohol misuse to 34% for anxiety/depression among current elite athletes, and from 16% for distress to 26% for anxiety/depression among former elite athletes [1]. These prevalence rates appeared comparable to or higher than those of non-athletes [1].

Since then, the IOC has undertaken several initiatives to translate the first IOC consensus statement and its related recommendations into practice within elite sport and to address remaining gaps in the field. Consequently, there has been an increasing number of epidemiological studies on mental health symptoms in elite athletes published since the systematic review and meta-analysis in 2019. Additionally, more attention has been given to the mental health symptoms reported by athletes’ entourage members (e.g., medical and performance staff) [4,5,6,7]. Initial research showed that performance staff of elite athletes report a prevalence of psychological distress similar to the prevalence among elite athletes, indicating that this group is also vulnerable to the pressure of high-performance sport environments [4,5,6,7]. With regard to (i) the large number of studies published in recent years on mental health symptoms in elite athletes and (ii) the emerging evidence of the mental symptoms reported by the athletes’ entourage members, an update of our meta-analysis is warranted. Furthermore, to the authors’ knowledge, no review of the available evidence on the mental health symptoms reported by both elite athletes and entourage members has been compiled. Such a review would support the 2026 update to the IOC consensus statement on mental health in elite athletes by contributing to the evidence synthesized in that statement.

Therefore, building on our systematic review and meta-analysis in 2019, the primary aim of our study was to review the existing epidemiological evidence on the prevalence of mental health symptoms among current and former elite athletes as well as among their entourage members. The secondary aim of our study was to compare the prevalence of mental health symptoms across groups.

## 2. Materials and Methods

A systematic review of the scientific literature was conducted and reported in accordance with PRISMA (Preferred Reporting Items for Systematic Reviews and Meta-Analyses) guidelines [8]. This study was preregistered (Open Science Framework ID: 9m86b).

### 2.1. Data Sources and Search Strategy

Several electronic databases, namely PubMed, PsycINFO, SportDiscus and Scopus, were searched from database inception until July 2025, and results were uploaded into Endnote 20. Duplicates were removed, and a final list of unique citations was distributed to topic members. An experienced academic librarian (MEH) developed a sensitive systematic search strategy based on three groups (concepts) of keywords and related synonyms: ‘mental health disorders’, ‘elite athletes’, and ‘entourage’ (Supplementary Material S1). Within each group, synonyms or related terms were linked using the Boolean command “OR” to expand the search, and then the Boolean command “AND” was used to combine the concepts to locate articles containing the search terms. Existing medical subject headings [MeSH] were used if possible.

### 2.2. Eligibility Criteria and Study Selection

The following eligibility criteria were used:

1. The study population consists of current or former elite athletes defined according to the IOC consensus statement on mental health in elite athletes in 2019 (being collegiate, Olympic, or professional athletes for current athletes and having competed as Olympic, or professional athletes for former athletes) and/or their entourage members (e.g., physiotherapists, nutritionists, coaches, strength and conditioning coaches, sports psychologists, sports medicine physicians, coaches) [2].
2. The study reports the prevalence of mental health symptoms (based on cut-off values).
3. The study is an original quantitative study (any study design).
4. The article is written in English.

After identifying and removing all duplicates, two researchers (VG and SCB) independently screened the titles and abstracts based on the defined criteria to identify potentially relevant articles. If the title and abstract did not provide enough information, the article was included for full-text review. Subsequently, two researchers (VG and SCB) independently assessed all full-text articles using the eligibility criteria. Additionally, references of the included studies were reviewed to ensure no relevant publications were overlooked. In case of disagreements regarding the inclusion or exclusion of an article, a third researcher (PG) was consulted.

### 2.3. Data Extraction

Two templates were created (one for elite athletes and one for entourage members) to extract data from the included articles. The following characteristics were extracted: study information (e.g., author); study design and related aspects (e.g., follow-up, response rate); study population (e.g., sample size, age, gender, country, type and level of sport); criteria and methods used to define and assess mental health symptoms; and the outcomes reported as prevalence. Two researchers (SCB and PG) extracted and cross-checked all included articles.

### 2.4. Risk of Bias

A 9-item tool designed for prevalence studies was utilized to assess the risk of bias in the included studies (Supplementary Material S2) [9]. All 9 potential bias domains were evaluated and rated as either a low (0) or high (1) risk of bias [9]. Studies were rated based on the cumulative score and categorized as follows: low risk of bias (scores ranging from 0 to 3); moderate risk of bias (scores ranging from 4 to 6); and high risk of bias (scores ranging from 7 to 9) [9]. The risk of bias for all included articles was evaluated by two researchers (SCB and VG). In case of discrepancies regarding their assessments, a third researcher (PG) was consulted.

### 2.5. Analysis

For our primary aim, the random-effects model was applied to all meta-analyses to account for between- and within-study variances, Meta-analyses were only performed if more than 5 original studies for a consistent population (e.g., current elite athletes, former elite athletes, current women elite athletes, former men elite athletes) were available to secure sufficient power, to offer greater robustness, to improve the reliability of between-study variance estimates and to allow for a more informative assessment of heterogeneity [10,11,12]. These statistical analyses were conducted separately for each group using OpenMetaAnalyst [13]. For the prevalence of mental health symptoms, pooled estimates with 95% confidence intervals (CIs) were calculated. Publication bias was assessed by visual inspection of the funnel plots. Variance between studies was assessed using Cochran’s Q and reported as I^2^. For our secondary aim, differences in prevalence between groups (if pooled estimates available) were examined using a 2-proportion z-test to evaluate whether the proportions in each group differed significantly (*p* < 0.05).

## 3. Results

### 3.1. Search Strategy

A total of 975 potentially relevant citations were identified. After removing 67 duplicates, 908 citations remained. After applying the inclusion criteria to the titles and abstracts, 111 potentially relevant studies were included for full-text review. Of these, 64 studies were excluded for various reasons (e.g., not in English, not an original study, not including relevant population, no prevalence reported). The reference check of the included studies identified 31 additional relevant studies. No disagreements between researchers regarding the inclusion or exclusion of articles were observed. Ultimately, our systematic review includes 72 original studies (PRISMA flow chart of the search procedure is provided in Supplementary Material S3).

### 3.2. Risk of Bias

Seventy of the 72 original studies included had an overall low risk of bias, and two studies had a moderate risk of bias (Supplementary Material S4).

### 3.3. Mental Health Symptoms Among Elite Athletes

Sixty-seven studies reporting mental health symptoms among elite athletes were included (Supplementary Material S5): 51 included solely current elite athletes [14,15,16,17,18,19,20,21,22,23,24,25,26,27,28,29,30,31,32,33,34,35,36,37,38,39,40,41,42,43,44,45,46,47,48,49,50,51,52,53,54,55,56,57,58,59,60,61,62,63,64], nine included solely former elite athletes [65,66,67,68,69,70,71,72,73] and seven included both current and former elite athletes [74,75,76,77,78,79,80]. The prevalence data presented in these included studies was across various sports (mostly team sports) and principally related to distress, depression, anxiety, sleep disturbance, alcohol misuse, drug misuse and disordered eating. All pooled estimates (if available) for the prevalence of mental health symptoms in elite athletes (per group) are presented in Table 1.

**Figure 1:**
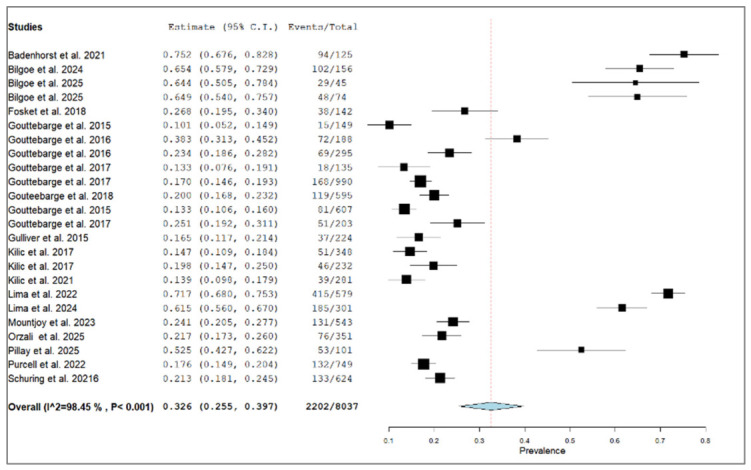
Random effects meta-analysis forest plot of the prevalence of distress in current elite athletes [16,21,22,28,32,33,34,35,36,38,46,47,49,51,53,55,74,75,76,77,78,79,80].

**Figure 2:**
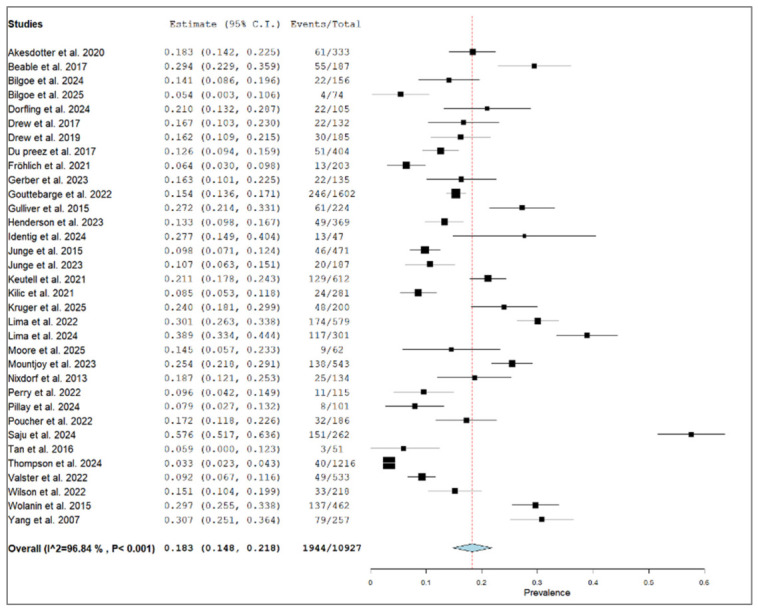
Random effects meta-analysis forest plot of the prevalence of depression in current elite athletes [15,18,22,23,24,25,26,29,30,37,38,40,41,42,43,44,45,46,47,50,52,53,54,56,58,59,61,62,63,64,74,79].

**Figure 3:**
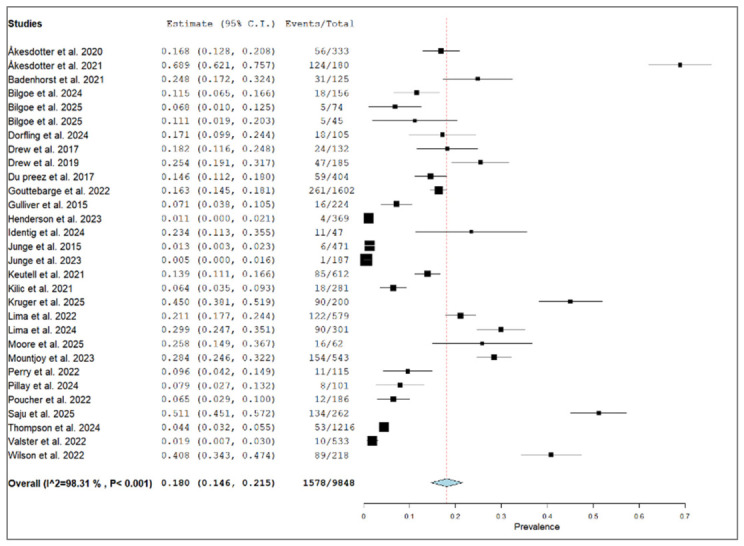
Random effects meta-analysis forest plot of the prevalence of anxiety in current elite athletes [14,15,16,21,22,23,24,25,26,37,38,40,41,42,43,44,45,46,47,48,49,52,53,54,56,59,61,62,74,79].

**Figure 4:**
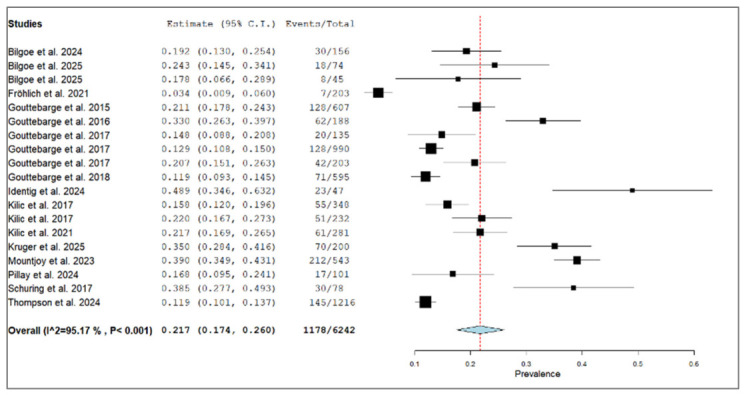
Random effects meta-analysis forest plot of the prevalence of sleep disturbance in current elite athletes [21,22,29,32,33,34,35,36,41,45,49,53,59,74,75,76,77,78,79,80].

**Figure 5:**
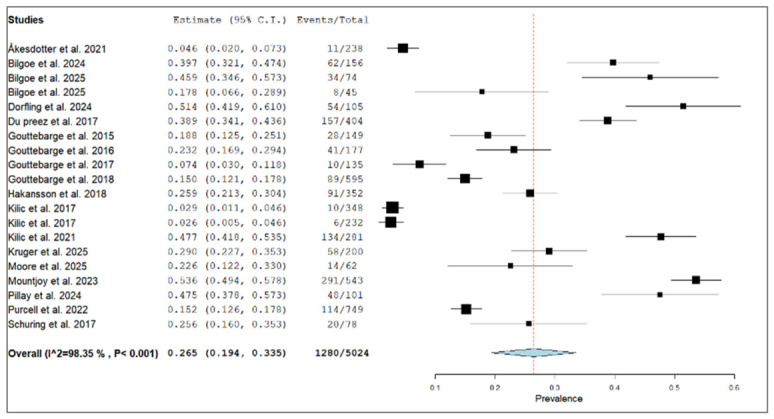
Random effects meta-analysis forest plot of the prevalence of alcohol misuse in current elite athletes [15,21,22,23,26,32,33,34,35,36,39,45,48,49,53,55,74,75,76,77,78,79,80].

**Figure 6:**
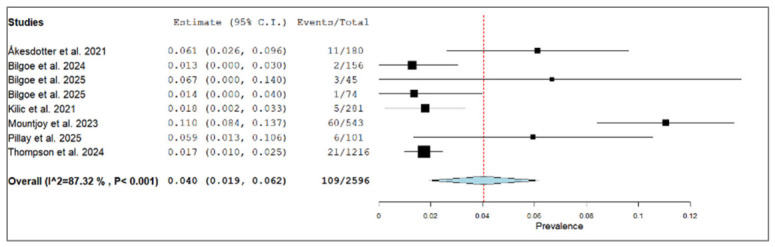
Random effects meta-analysis forest plot of the prevalence of drug misuse in current elite athletes [14,21,22,49,53,59,74,79].

**Figure 7:**
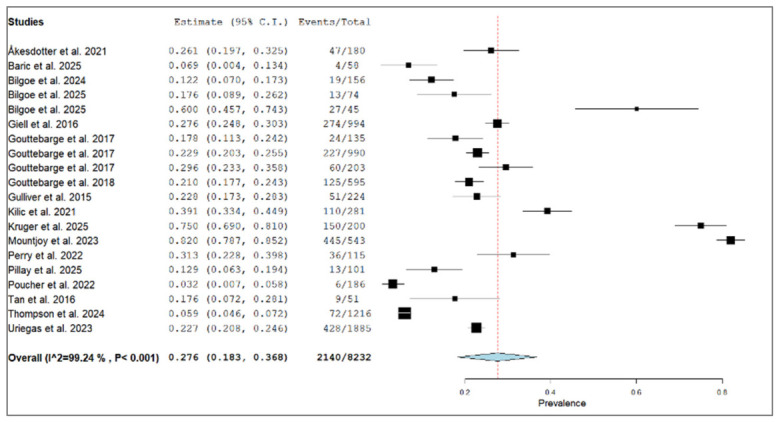
Random effects meta-analysis forest plot of the prevalence of disordered eating in current elite athletes [14,17,21,22,31,32,35,36,38,45,49,53,54,58,59,60,74,76,77,79].

For our meta-analyses of current elite athletes, 24 studies covering 8037 athletes were used to estimate the pooled prevalence of distress. Our meta-analysis (Figure 1; Table 1) showed that 32.6% (95% CI 25.5–39.7) of current elite athletes reported symptoms of distress (considerable heterogeneity: Q = 1485.946, *p* < 0.001, I2 = 98.45%). Thirty-five studies reported prevalence data on depressive symptoms among 10,927 current elite athletes. Our meta-analysis (Figure 2; Table 1) revealed that 18.3% (95% CI 14.8–21.8) of current elite athletes reported symptoms of depression (considerable heterogeneity: Q = 1043.655, *p* < 0.001, I2 = 96.84%). Thirty studies among 9848 current elite athletes were used for the pooled prevalence of anxiety. Our meta-analysis (Figure 3; Table 1) showed that 18.0% (95% CI 14.6–21.5) of current elite athletes reported symptoms of anxiety (considerable heterogeneity: Q = 1718.202, *p* < 0.001, I2 = 98.31%). Nineteen studies reported prevalence data on sleep disturbance among 6242 current elite athletes. Our meta-analysis (Figure 4; Table 1) revealed that 21.7% (95% CI 17.4–26.0) of current elite athletes reported sleep disturbance (considerable heterogeneity: Q = 372.296, *p* < 0.001, I2 = 95.17%). Twenty studies among 5024 current elite athletes were used for the pooled prevalence of alcohol misuse. Our meta-analysis (Figure 5; Table 1) showed that 26.5% (95% CI 19.4–33.5) of current elite athletes reported alcohol misuse (considerable heterogeneity: Q = 1151.217, *p* < 0.001, I2 = 98.35%). Eight studies reported prevalence data on drug misuse among 2596 current elite athletes. Our meta-analysis (Figure 6; Table 1) revealed that 4.0% (95% CI 1.9–6.2) of current elite athletes reported drug misuse (considerable heterogeneity: Q = 55.185, *p* < 0.001, I2 = 87.32%). Twenty studies covering 8232 current elite athletes were used to estimate the pooled prevalence of disordered eating. Our meta-analysis (Figure 7; Table 1) showed that 27.6% (95% CI 18.3–36.8) of current elite athletes reported disordered eating (considerable heterogeneity: Q = 2496.711, *p* < 0.001, I2 = 99.24%).

For current men elite athletes, up to 24 studies covering up to 5533 men athletes were used for our meta-analysis. The pooled prevalence was 28.0% (95% CI 17.3–42.0) for distress, 18.3% (95% CI 13.3–24.6) for depression, 14.9% (95% CI 10.8–20.2) for anxiety, 11.8% (95% CI 5.9–22.1) for sleep disturbance, 43.7% (95% CI 41.2–46.3) for alcohol misuse and 19.4% (95% CI 18.1–20.7) for disordered eating.

For current elite women athletes, up to 22 studies covering up to 3027 women athletes were included in our meta-analysis. The pooled prevalence was 34.7% (95% CI 32.0–37.4) for distress, 19.8% (95% CI 18.4–21.2) for depression, 18.6% (95% CI 17.1–20.0) for anxiety, 28.8% (95% CI 26.0–31.5) for sleep disturbance, 40.2% (95% CI 24.4–58.3) for alcohol misuse and 34.6% (95% CI 24.7–45.9) for disordered eating.

**Figure 8:**
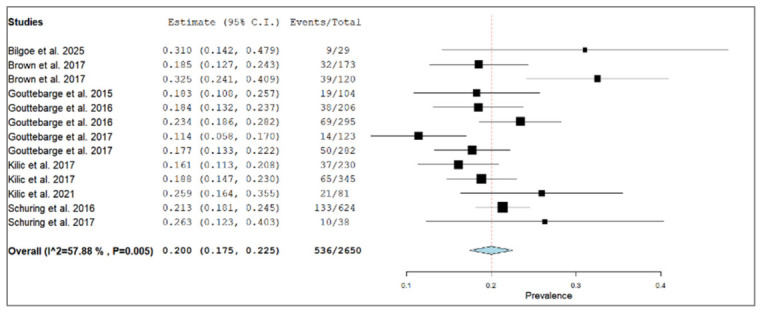
Random effects meta-analysis forest plot of the prevalence of distress in former elite athletes [65,66,67,71,74,75,76,77,78,79,80].

**Figure 9:**
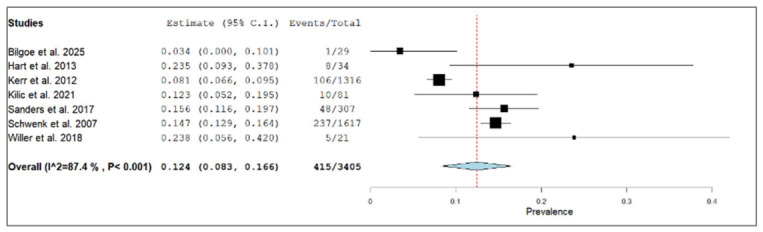
Random effects meta-analysis forest plot of the prevalence of depression in former elite athletes [68,69,70,72,73,74,79].

**Figure 10:**
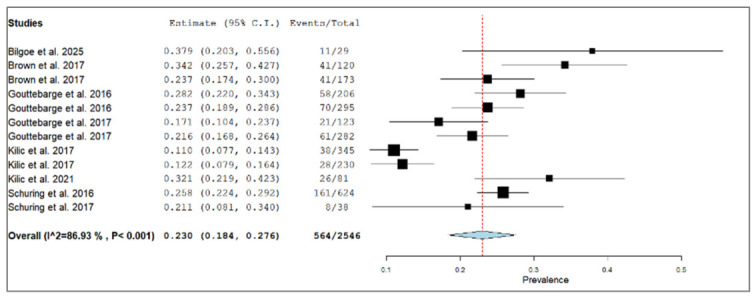
Random effects meta-analysis forest plot of the prevalence of sleep disturbance in former elite athletes [65,66,67,71,74,76,77,78,79,80].

**Figure 11:**
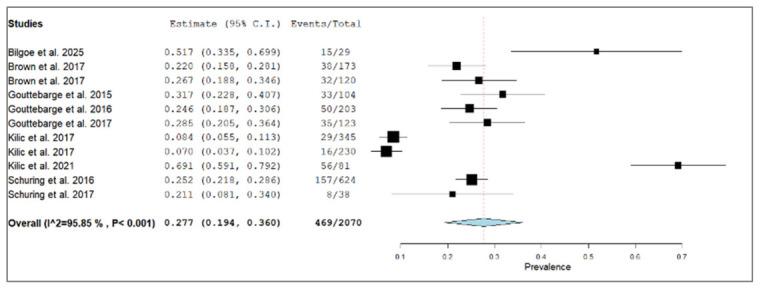
Random effects meta-analysis forest plot of the prevalence of alcohol misuse in former elite athletes [65,66,67,71,74,75,78,79,80].

For our meta-analyses of former elite athletes, 13 studies covering 2650 athletes were used for the pooled prevalence of distress. Our meta-analysis (Figure 8; Table 1) showed that 20.0% (95% CI 17.5–22.5) of former elite athletes reported symptoms of distress (moderate heterogeneity: Q = 28.493, *p* < 0.005, I2 = 57.88%). Seven studies reported prevalence data on depressive symptoms among 3405 former elite athletes. Our meta-analysis (Figure 9; Table 1) revealed that 12.4% (95% CI 8.3–16.6) of former elite athletes reported symptoms of depression (considerable heterogeneity: Q = 47.624, *p* < 0.001, I2 = 87.4%). Twelve studies covering 2546 former elite athletes were used to estimate the pooled prevalence of sleep disturbance. Our meta-analysis (Figure 10; Table 1) showed that 23.0% (95% CI 18.4–27.6) of former elite athletes reported sleep disturbance (considerable heterogeneity: Q = 84.177, *p* < 0.001, I2 = 86.93%). Eleven studies reported prevalence data on alcohol misuse among 2070 former elite athletes. Our meta-analysis (Figure 11; Table 1) revealed that 27.7% (95% CI 19.4–36.0) of former elite athletes reported alcohol misuse (considerable heterogeneity: Q = 240.758, *p* < 0.001, I2 = 95.85%).

For former men elite athletes, up to 9 studies covering up to 3376 men athletes were included in our meta-analysis. The pooled prevalence was 21.8% (95% CI 16.8–27.8) for distress, 14.2% (95% CI 10.2–19.4) for depression, 23.8% (95% CI 19.2–29.0) for sleep disturbance and 25.6% (95% CI 16.8–36.9) for alcohol misuse.

### 3.4. Mental Health Symptoms Among Entourage Members

Seven studies reporting mental health symptoms among entourage members were included (Supplementary Material S6), six of which were conducted among high-performance staff (including coaches). The prevalence data presented in these included studies was collected across various sports (mostly team sports) and principally related to distress, depression, anxiety, sleep disturbance, alcohol misuse, drug misuse and disordered eating. Because of the limited number of studies, no meta-analysis was performed.

The included studies showed that the prevalence of mental health symptoms in high-performance staff (including coaches) ranged from 5% for depression and anxiety to 53% for alcohol misuse [4,14,22,81,82,83]. A study among healthcare professionals working during the Paralympic games showed a prevalence of mental health symptoms reaching 12% for depression, 8% for anxiety, and 36–58% for burnout [6]. Among these healthcare professionals, 8% reported thoughts of self-harm or suicidal thoughts a few days per month.

### 3.5. Differences in Prevalence Between Groups

A comparison in prevalence between current and former elite athletes was performed for four mental health symptom clusters, namely distress, depression, sleep disturbance and alcohol misuse. Current elite athletes showed a significantly higher prevalence of distress (32.6% vs. 20.0%; *p* < 0.05) and depression (18.3% vs. 12.4%; *p* < 0.05) compared to former elite athletes. For sleep disturbance (21.7% vs. 23.0%) and alcohol misuse (26.5% vs. 27.7%), no statistically significant differences were found between current and former elite athletes.

Comparison in prevalence between current men and women elite athletes was performed for five mental health symptom clusters, namely distress, depression, anxiety, sleep disturbance and alcohol misuse. Current women elite athletes showed a significantly higher prevalence of distress (34.7% vs. 28.0%; *p* < 0.05), anxiety (18.6% vs. 14.9%; *p* < 0.05) and sleep disturbance (28.8% vs. 11.8%; *p* < 0.05) compared to current men elite athletes.

For depression (18.3% vs. 19.8%) and alcohol misuse (43.7% vs. 40.2%), no statistically significant differences were found between current men and women elite athletes.

## 4. Discussion

We reviewed the existing epidemiological evidence regarding the prevalence of mental health symptoms and disorders in elite athletes and their entourage members. We retrieved a total of 72 original studies (65 on elite athletes, 5 on entourage members, and two covering both groups). Meta-analyses comprising up to 10,927 current elite athletes showed that the prevalence of mental health symptoms ranged from 4% for drug misuse to 33% for distress, being generally more reported by women athletes. Meta-analyses comprising up to 3405 former elite athletes showed that the prevalence of mental health symptoms ranged from 12% for depression to 28% for alcohol misuse. Because of the considerable heterogeneity of included studies, the pooled prevalence estimates from these meta-analyses should be interpreted cautiously, as they summarize highly heterogeneous populations. Mental health symptoms were found to be also reported by entourage members, with prevalence rates ranging from 5% for depression or anxiety to 53% for alcohol misuse in high-performance staff (including coaches) and reaching up to 36–58% for burnout in healthcare professionals working during the Paralympic games.

### 4.1. Exponential Increase in Evidence Among Elite Athletes

The number of epidemiologic studies exploring the prevalence of mental health symptoms among current and former elite athletes seems to have increased in recent years. In comparison to our 2019 manuscript, the number of original studies included in our systematic review and meta-analysis has doubled (34 vs. 67) [1]. While several steps were used to strengthen our rigorous methodological process (e.g., inclusion criteria, risk of bias), having a higher number of original studies to use in our meta-analysis has various benefits: (i) it strengthens the overall reliability, validity, robustness and generalizability of our findings and conclusions, (ii) it increases the statistical power, as more data points enable identification of true precise estimates, and (iii) it reduces the influence of random variation and the impact of any single study’s bias. Another positive evolution since our 2019 manuscript is the increased consistency in the use of similar validated questionnaires across studies. In 2020, the IOC published its Sport Mental Health Assessment Tool 1 (SMHAT-1) specifically compiled to assess elite athletes potentially at risk for or already experiencing mental health symptoms and disorders [84]. The IOC SMHAT-1 is largely based on seven validated questionnaires exploring seven mental health symptom clusters. Unsurprisingly, these seven questionnaires have been regularly used (in whole or part) since publication of the IOC SMHAT-1 in empirical research within the elite sport ecosystem (14 studies). This has increased the homogeneity of the data collected and enables more valid comparisons between studies and sports, as well as between current and former elite athletes.

Our meta-analyses revealed that the prevalence of distress and depression was higher in current than in former elite athletes. In addition to non-sport factors, such as adverse life events, current elite athletes are also exposed to sport-specific stressors during their career, e.g., severe musculoskeletal injuries leading to prolonged periods without training and competition or decreased sport performance [2,85,86]. This might explain the higher prevalence found in current elite athletes. By contrast, the prevalence of sleep disturbance and alcohol misuse calculated in our meta-analyses was similar among current and former elite athletes. Regarding alcohol misuse, this finding was unexpected, as we anticipated that alcohol misuse would be less common among current elite athletes than among former athletes, given the detrimental effects of alcohol on performance and recovery [2,85]. One possible explanation for this comparable prevalence of alcohol misuse is that alcohol consumption might be culturally normative within certain sports. Additionally, even if current elite athletes do not misuse alcohol during their active competitive seasons, they may be particularly likely to do so during the off-season. Our meta-analyses also confirmed previous statements that the prevalence of mental health symptoms is higher in current women elite athletes than in men athletes [2]. Among former elite athletes, alcohol misuse might originate from the various challenges associated with transitioning out of sport [2,85]. In contrast to alcohol, drug misuse among current elite athletes seems less of a concern (prevalence of 4%). Our meta-analyses showed that nearly one out of four athletes (current or former) report sleep disturbance. This is a confirmation that many elite athletes do not sleep the daily recommended number of hours per night, likely due to factors such as pre-competition stress, late-evening sporting events, early-morning training sessions and travel. As sleep disturbance is likely to impair athletes’ performance and recovery, it remains essential to consider the implementation of interventions such as sleep hygiene education [87]. In the absence of reference groups from the general population in the studies included in our review, it remains challenging to put our findings in a broader perspective. However, our systematic review and meta-analyses suggest that the prevalence of mental health symptoms among current and former elite athletes is at least comparable to that observed in the general population and, in some cases, might even be higher [88].

The findings discussed in the previous section from our meta-analysis and related between-group differences should be interpreted with caution; these findings are based on studies with considerable heterogeneity and are derived from different study populations with different methodological characteristics. Additionally, as study-level confounding factors were not accounted for, our meta-analysis and related comparisons can be seen as indirect and, thus, cannot be interpreted as true estimates or evidence of true between-group differences.

### 4.2. Emerging Evidence Among Entourage Members

Even though more attention has recently been given to the mental health of athletes’ entourage members, the number of empirical studies conducted among high-performance staff (including coaches) and medical staff working in the elite sport ecosystem remains limited and, therefore, should be interpreted with caution. However, the emerging evidence as indicated in our review shows that the prevalence of mental health symptoms reached up to 53% for alcohol misuse in high-performance staff (including coaches) and 58% for burnout in healthcare professionals. This is alarming but not surprising, as high-performance staff (including coaches) and medical staff are required to work in highly demanding roles characterized by multifaceted stressors and pressures [89]. These multiple performance and non-performance related demands, including strategic development, athletes’ selection, management of other staff members, extensive travel and lack of a typical work-life balance, are even more pronounced during key competition events such as the Olympic and Paralympic games [89]. Our review emphasizes the need for further research about the mental health of athletes’ entourage members.

### 4.3. Strengths and Limitations

The three principal limitations of the studies included in our systematic review are that (1) nearly all relied on self-reported data, (2) a number of different instruments were used to assess mental health symptoms, and (3) the included studies exhibited considerable heterogeneity. Regarding the first, the studies included in our systematic review and meta-analyses primarily assessed self-reported mental health symptoms that are subject to selection and recall bias. Future research should also address clinically diagnosed mental health disorders defined according to existing diagnostic criteria, such as in the Diagnostic and Statistical Manual of Mental Disorders (DSM-5-TR) or International Classification of Diseases (ICD) [90]. Regarding the second limitation, even if there is less pronounced instrument heterogeneity than a few years ago, the instruments used to assess mental health symptoms, as well as the mental health symptom clusters under study, still show some variation across the included studies. Using heterogeneous instruments made it challenging to perform meta-analyses for a given mental health symptom cluster or with all included studies. It is also important to note that most of the instruments used were not specifically developed for elite athlete populations, such that their level of validity could be questioned. In recent years, the instruments embedded in steps 1 and 2 of the IOC SMHAT-1 were used across several sports and countries, which adds value when performing meta-analyses. Regarding the third limitation, the considerable heterogeneity among the included studies (e.g., objectives, types of samples, representativeness) might have affected the precision and generalizability of the pooled prevalence estimates. Beyond these three principal limitations, it is worth mentioning that research has largely concentrated on men elite athletes participating in team sports, which limits the validity of comparisons across sport types (e.g., individual versus team), regions and cultures (e.g., Asian versus African athletes), genders, and other demographic characteristics. Future investigations should therefore prioritize the inclusion of women elite athletes, both active and retired, as study participants, as well as para-athletes and athletes’ entourages. Finally, longitudinal investigations adopting prospective cohort designs should be conducted over a single sport season or ideally over several years. This would enable (i) elucidation of the temporal trajectory of mental health symptoms and disorders in elite sport and (ii) examination of causal associations between potential contributing factors and mental health symptoms and disorders.

### 4.4. Recommendations for Practice

Despite the growing body of scientific research on mental health symptoms in elite sports, the authors emphasize that raising awareness and prioritizing mental health within elite sport settings must be actively promoted by international and national stakeholders as well as by sport federations. Such a recommendation was already issued by an international think tank of the International Society of Sport Psychology in their consensus statement, urging sport organizations to embed athlete mental health within elite sport systems by supporting research, improving staff mental health literacy, and establishing dedicated mental health oversight [91]. This is crucial as the elite sport ecosystem continues to struggle with the persistent stigma surrounding mental health symptoms and disorders. This is even more the case in specific countries, regions and cultures. Given the prevalence of mental health symptoms among current elite athletes (which are greater among women athletes), the provision of interdisciplinary medical and psychological support should represent a minimum standard of care. In this regard, sports medicine physicians and licensed/registered mental health professionals play a pivotal role in ensuring early identification and appropriate management of mental health symptoms and disorders in elite athletes. Early identification could be facilitated by using the IOC Sport Mental Health Assessment Tool 2 (IOC SMHAT2) [92]. Timely diagnosis and adequate treatment are essential to optimize athletic performance, health and overall quality of life. Mental health support should also be directed towards former elite athletes as indicated in our review. Such support could be operationalized as an end-of-career assessment as has recently been piloted within the After Career Consultation in elite football and rugby [93,94]. This support is likely to be a valuable approach with positive outcomes for former elite athletes, especially for those facing involuntary retirement. Comprehensive preparation for post-sport life could also mitigate the risk of mental health symptoms among former elite athletes, for example through targeted education programs, resource centers, and training in mental and life skills to support career transitions. The aforementioned resources, in a tailored format, should also be made available to the high-performance (including coaches) and medical staff working in the elite sport ecosystem.

## 5. Conclusions

Showing considerable heterogeneity across included studies, our systematic review and meta-analysis established that mental health symptoms are commonly reported by current and former elite athletes, as well as by their high-performance staff (including coaches) and medical staff. This warrants the implementation of various tailored resources, such as mental health literacy, screening programs and interdisciplinary medical and psychological support.

## Figures and Tables

**Table 1:** Prevalence (%) and 95% confidence intervals for mental health symptoms among elite athletes (total, men, women).

Mental Health Symptom	Cluster	Total	Men	Women
**Distress**	Current elite athletes	32.6 (25.5–39.7)	28.0% (17.3–42.0)	34.7 (32.0–37.4)
	Former elite athletes	20.0 (17.5–22.5)	21.8 (16.8–27.8)	NA
**Depression**	Current elite athletes	18.3 (14.8–21.8)	18.3 (13.3–24.6)	19.8 (18.4–21.2)
	Former elite athletes	12.4 (8.3–16.6)	14.2 (10.2–19.4)	NA
**Anxiety**	Current elite athletes	18.0 (14.6–21.5)	14.9 (10.8–20.2)	18.6 (17.1–20.0)
	Former elite athletes	NA	NA	NA
**Sleep disturbance**	Current elite athletes	21.7 (17.4–26.0)	11.8 (5.9–22.1)	28.8 (26.0–31.5)
	Former elite athletes	23.0 (18.4–27.6)	23.8 (19.2–29.0)	NA
**Alcohol misuse**	Current elite athletes	26.5 (19.4–33.5)	43.7 (41.2–46.3)	40.2 (24.4–58.3)
	Former elite athletes	27.7 (19.4–36.0)	25.6 (16.8–36.9)	NA
**Drug misuse**	Current elite athletes	4.0 (1.9–6.2)	NA	NA
	Former elite athletes	NA	NA	NA
**Disordered eating**	Current elite athletes	27.6 (18.3–36.8)	19.4 (18.1–20.7)	34.6 (24.7–45.9)
	Former elite athletes	NA	NA	NA

NA, not applicable (<6 studies available).

## Data Availability

All data presented in the article are available (including online Supplementary Materials).

## Supplementary Materials

The following supporting information can be downloaded at https://www.mdpi.com/article/10.3390/sports14070296/s1, S1: Search strategy; S2: Risk of bias criteria; S3: Flow chart of the search procedure; S4: Risk of bias of the included studies; S5: Prevalence of mental health symptoms among current and former elite athletes: data extraction from included studies; S6: Prevalence of mental health symptoms among entourage members: data extraction from included studies; PRISMA 2020 Checklist.
