# Supplementary material for: Supporting the IOC Consensus Statement on Mental Health in Elite Athletes: A Systematic Review and Meta-Analysis on the Prevalence of Mental Health Symptoms in Elite Sports
Source: Sports (Basel). 2026 Jul 10;14(7):296. doi: 10.3390/sports14070296 (PMC13417594; doi:10.3390/sports14070296)
Supplement: Supplementary file 1 [file sports-14-00296-s001.zip › sports-4388869-supplementary.pdf]

Supplementary material S1: Search strategy

PubMed (Medline)

((Olympic*[tiab] OR "high level"[tiab] OR "highly trained"[tiab] OR collegiate[tiab] OR "NCAA"[tiab] OR elite[tiab] OR professional[tiab] OR college[tiab] OR "Universities"[Mesh]) AND ("Sports"[Mesh] OR sports[tiab] OR athletic[tiab])) AND ("Mental Health"[Mesh] OR "Mental Disorders"[Mesh] OR "mental health"[tiab] OR "mental disorder*"[tiab] OR "mental illness*"[tiab] OR psychiatric[tiab] OR "severe mental"[tiab] OR psychotherapy[tiab] OR depression[tiab] OR anxiety[tiab] OR "mood disorder*"[tiab] OR wellbeing[tiab] OR well-being[tiab])) AND (coach*[title] OR entourage[title] OR staff[title])

PsycINFO (Ebsco)

((DE "Coaches")) OR TI (staff OR coach* OR entourag*)) AND TI (well-being OR mental health or mental illness or mental disorder or psychiatric illness or anxiety or depression or well-being or distress) AND sports

Limited to Academic Journals only

SportDiscus (Ebsco)

(mental health or mental illness or mental disorder or psychiatric illness or anxiety or depression) AND TI (coach* OR staff OR entourage) AND (sport or athlete* or players OR olympic* OR paralymp*)

Scopus (Elsevier)

TITLE(athlete* OR sport* OR athletic* OR paralymp* OR Olympi* OR player OR coach* OR staff OR entourage) AND TITLE({mental health} OR {mental illness} OR depression OR psychiatric OR psychological OR psychotic OR {mood disorder} OR wellbeing OR stress OR well-being OR emotional) AND TITLE(prevalence OR incidence OR frequenc* OR rates OR occurrence OR trends OR statistical))

Online supplementary material S2: Risk of bias criteria

1. Was the study's target population a close representation of the national population in relation to relevant variables, e.g. age, sex, occupation? (Yes = 0; No = 1)

2. Was the sampling frame a true or close representation of the target population? (Yes = 0; No = 1)

3. Was some form of random selection used to select the sample, OR, was a census undertaken? (Yes = 0; No = 1)

4. Was the likelihood of non-response bias minimal? (Yes = 0; No = 1)

5. Were data collected directly from the subjects (as opposed to a proxy)? (Yes = 0; No = 1)

6. Was an acceptable case definition used in the study? (Yes = 0; No = 1)

7. Was the study instrument that measured the parameter of interest (e.g. prevalence of low back pain) shown to have reliability and validity (if necessary)? (Yes = 0; No = 1)

8. Was the same mode of data collection used for all subjects? (Yes = 0; No = 1)

9. Were the numerator(s) and denominator(s) for the parameter of interest appropriate? (Yes = 0; No = 1)

Supplementary material S3: Flow chart of the search procedure

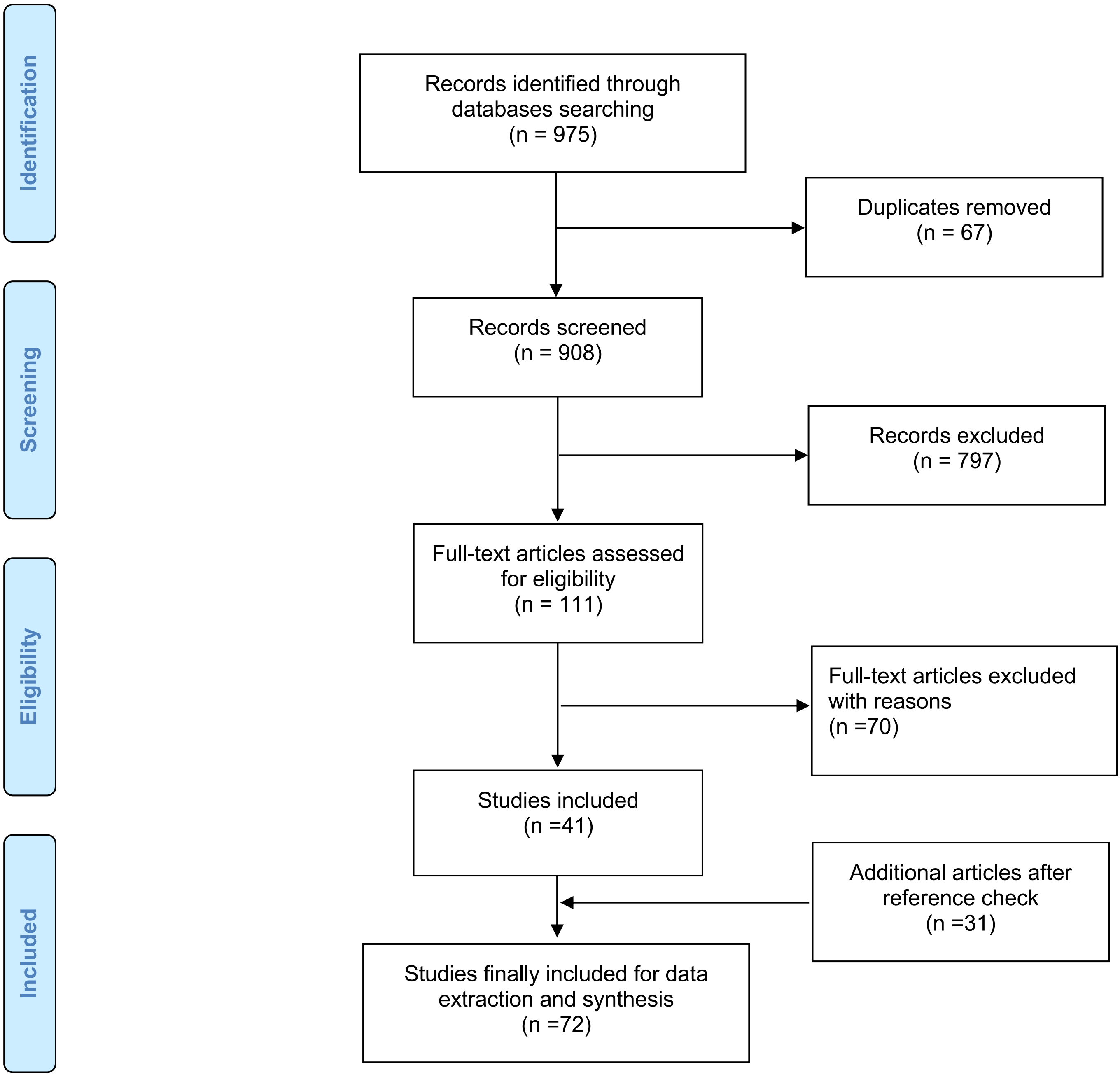

Supplementary material S4: Risk of bias of the included studies

Study	1	2	3	4	5	6	7	8	9	Risk of bias
Åkesdotter 2022 [14]	0	0	0	u	0	0	0	1	0	Low
Åkesdotter 2020 [15]	0	0	0	1	0	0	0	0	0	Low
Badenhorst 2021 [16]	0	0	0	1	0	0	0	0	0	Low
Barić 2024 [17]	0	0	0	u	0	0	0	0	0	Low
Beable 2017 [18]	0	0	0	1	0	0	0	0	0	Low
Benjamin 2020 [19]	0	0	0	u	0	0	0	0	0	Low
Biggins 2018 [20]	0	0	0	1	0	0	0	0	0	Low
Bilgoe 2025 [74]	0	0	1	1	0	0	0	0	0	Low
Bilgoe 2024 [21]	0	0	0	u	0	0	0	0	0	Low
Bilgoe 2024 [22]	0	0	0	0	0	0	0	0	0	Low
Brown 2017 [65]	0	0	0	1	0	0	0	0	1	Low
Dorfling 2024 [23]	0	0	0	1	0	0	0	0	0	Low
Drew 2019 [25]	0	0	1	1	0	0	0	1	0	Low
Drew 2018 [24]	0	0	0	1	0	0	0	0	0	Low
Du Preez 2017 [26]	0	0	0	1	0	0	0	0	0	Low
Eken 2023 [6]	0	0	0	1	0	0	0	0	0	Low
Escobar-Molina 2015 [27]	0	0	0	U	0	0	0	0	0	Low
Foskett 2018 [28]	0	0	0	U	0	0	0	0	0	Low
Fröhlich 2021 [29]	0	0	0	u	0	0	0	0	0	Low
Gerber 2023 [30]	0	0	1	1	0	0	0	0	0	Low
Giel 2016 [31]	0	0	0	1	0	0	0	0	1	Low
Gorczynski 2020 [81]	1	1	1	u	0	0	0	0	0	Moderate
Gouttebarge 2015 [33]	0	0	0	1	0	0	0	0	0	Low
Gouttebarge 2015 [75]	0	0	0	1	0	0	0	0	0	Low
Gouttebarge 2016 [34]	0	0	0	1	0	0	0	0	0	Low
Gouttebarge 2016 [66]	0	0	0	1	0	0	0	0	0	Low
Gouttebarge 2016 [67]	0	0	0	1	0	0	0	0	0	Low

Gouttebarge 2017 [32]	0	0	0	1	0	0	0	0	1	Low
Gouttebarge 2017 [35]	0	0	0	1	0	0	0	0	1	Low
Gouttebarge 2017 [77]	0	0	0	1	0	0	0	0	1	Low
Gouttebarge 2017 [76]	0	0	0	1	0	0	0	0	1	Low
Gouttebarge 2018 [36]	0	0	0	1	0	0	0	0	1	Low
Gouttebarge 2022 [37]	0	0	0	0	0	0	0	0	0	Low
Gulliver 2015 [38]	0	0	0	1	0	0	0	0	1	Low
Hakansson 2018 [39]	0	0	0	1	0	0	0	0	0	Low
Hart 2013 [68]	0	0	0	0	0	0	0	0	0	Low
Henderson 2023 [40]	0	0	1	0	0	0	0	0	0	Low
Identeg 2024 [41]	0	1	1	u	0	0	0	0	0	Low
Junge 2023 [43]	0	0	1	0	0	0	0	0	0	Low
Junge 2016 [42]	0	0	1	U	0	0	0	1	0	Low
Kegelaers 2021 [82]	0	0	0	1	0	0	0	0	0	Low
Kerr 2012 [69]	0	0	0	1	0	1	1	0	0	Low
Kim 2020 [83]	0	0	0	1	0	0	0	0	0	Low
Kilic 2017 [78]	0	0	0	1	0	0	0	0	1	Low
Kilic 2021 [79]	0	0	0	u	0	0	0	0	0	Low
Kruger 2022 [45]	1	1	1	u	0	0	0	0	0	Low
Kuettel 2021 [44]	0	0	1	u	0	0	0	0	0	Low
Lima 2022 [46]	1	0	0	u	0	0	0	0	0	Low
Lima 2024 [47]	0	0	0	1	0	0	0	0	0	Low
Moore 2025 [48]	0	0	0	1	0	0	0	0	0	Low
Mountjoy 2023 [49]	0	0	0	1	0	0	0	0	0	Low
Nixdorf 2013 [50]	0	1	0	u	0	0	0	0	0	Low
Orzali 2025 [51]	0	0	0	0	0	0	0	0	0	Low
Perry 2022 [52]	0	1	1	1	0	0	0	0	0	Low
Pilkington 2022 [4]	0	0	0	u	0	0	0	0	0	Low
Pillay 2024 [53]	0	0	1	0	0	0	0	0	0	Low

Poucher 2022 [54]	0	1	1	u	0	0	0	0	0	Low
Purcell 2020 [55]	0	0	0	u	0	0	0	0	0	Low
Saju 2025 [56]	0	0	1	1	0	0	0	0	0	Low
Sanders 2017 [70]	1	1	0	1	0	0	0	0	0	Low
Schaal 2011 [57]	0	0	0	U	0	0	0	0	0	Low
Schuring 2017 [80]	0	0	0	1	0	0	0	0	1	Low
Schuring 2017 [71]	0	0	1	1	0	0	0	0	0	Low
Schwenk 2007 [72]	0	0	0	1	0	1	0	0	1	Low
Tan 2016 [58]	0	0	0	1	0	1	0	0	1	Low
Thompson 2024 [59]	0	0	0	0	0	0	0	0	0	Low
Uriegas 2023 [60]	0	1	0	u	0	0	0	0	0	Low
Valster 2022 [61]	0	0	0	0	0	0	0	0	0	Low
Willer 2018 [73]	0	1	1	U	0	0	0	0	1	Moderate
Wilson 2022 [62]	0	0	0	u	0	0	0	0	0	Low
Wolanin 2016 [63]	0	0	0	1	0	0	0	0	0	Low
Yang 2007 [64]	0	1	1	0	0	0	0	0	0	Low

U, unclear

Supplementary material S5: Prevalence of mental health symptoms among current and former elite athletes: data extraction from included studies

Authors (year)	Study population	Study design	Outcome measurements (symptoms, instruments, threshold)	Prevalence %
Akesdotter et al. (2020) [15]	C: Sweden<br>A: 24.6±3.6<br>N: 333<br>G: 136.86/196.14<br>R/E%:NP<br>SO:NP<br>J: Current elite athletes<br>S: Basketball, athletics, golf , gymnastics, handball, canoe, cross-country skiing, orienteering, equestrian, sailing, swimming, alpine skiing, power lifting	Cross-sectional	Definition: Prevalence of mental health problems: symptoms of depression, anxiety, alcohol misuse, ADHD, and symptoms of burnout (RA:reduced sense of acomplishment, EXH: emotional/physical exhaustion, DEV: sport devaluation)<br><br>Instrument(s):<br>- Depression: PHQ-9 cut-off (≥ 10)<br>- Anxiety: GAD-7 cut-off (≥ 10)<br>- alcohol misuse: AUDIT-C, cut-off (≥ 5)<br>- Symptoms of ADHD: ASRS-V1.1, cut- off (≥ 24)<br>- Burnout: Athlete burnout questionnaire ABQ RA, EXH, DEV, Range (5-25)	- Depression: 18.3% (n=61)<br>- Anxiety: 16.8% (n=56)<br>- alcohol misuse: 25.8% (n=86)<br>- Symptoms of ADHD: 5.4% (n=18)<br>- Burnout, (mean value): ABQ RA (12.78(±4.30)), EXH (12.05 (±4.11)), DEV (10.83(±4.46))
Åkesdotter et al. (2022) [14]	C: Sweden<br>A: 23.5±5.9<br>N: 180<br>G: : 58/122<br>R/E%:NP<br>SO:NP<br>J: Current elite athletes<br>S: Various	Descriptive observational design	Definition: Prevalence of psychiatric disorders, namely affective disorders, anxiety disorders, eating disorders, disorders due to psychoactive substance use, alcohol-related disorders<br><br>Instrument(s): diagnostic interviews based on the following:<br>- Any affective disorder: ICD-10:F30-F39	Any affective disorder: total 51% (n=180), Female 50% (n=122), male 52% (n=58)<br>- Any Anxiety disorder : total 69% (n=180), Female 70% (n=122), male 67% (n=58)<br>- Any Eating disorder: total 26% (n=180), Female 37% (n=122), male n<5 (n=58)<br>- Any disorder due to psychoactive substance use: total 6% (n=180), Female n<5 (n=122), male 14% (n=58)

			- Any Anxiety disorder : ICD-10: F40-F43<br>- Any Eating disorder: ICD-10: F50<br>- Any Disorder due to psychoactive substance use:<br>- Alcohol-related disorders: F10,Z721	- Alcohol-related disorders: total 6% (n=180), Female n<5 (n=122), male 14% (n=58)
Badenhorst et al. (2021)<br>[16]	C: South Africa<br>A: 26.7 ± 9.2<br>N: 125<br>G: 94/31<br>R/E%:NP<br>SO:NP<br>J: Elite Para-athletes<br>S: Boccia, football-7-a-side, goalball, para athletics, para cycling, para judo, para powerlifting, para swimming.	Cross-sectional	Definition: Prevalence of symptoms of psychological distress and anxiety<br>Instrument(s):<br>- Distress: K-10, cut-off (≥16)<br>- Anxiety: STAI-T, cut-off (≥45)	- Distress: 76% (n=94)<br>- Anxiety: 25% (n=31)
Baric et al. (2024)<br>[17]	C: Croatia<br>A: 14.01 ± 3.06<br>N: 58<br>G: 25/33<br>R/E%:NP<br>SO:NP<br>J: Current elite athletes<br>S: Taekwondo	Cross-sectional	Definition: Prevalence of symptoms of eating disorders.<br><br>Instrument(s):<br>- Disordered eating: EAT-26(≥ 20)	Disordered eating:<br>Male 0% (0)<br>Female 12.1% (4)
Beable et al. (2017)<br>[18]	C: New Zealand<br>A: 24.5±4.4<br>N: 187<br>G: 74/113<br>R/E%:NP<br>SO:NP<br>J: Current elite athletes<br>S: Various	Cross-sectional	Definition: Prevalence of symptoms of depression<br><br>Instrument(s):<br>- Depression: CESD-R (≥16)	- Depression: 29.6% (n=55)
Benjamin CL et al. (2020)<br>[19]	C: USA<br>A: 20.0± 2.0<br>N: 230<br>G: 110/120	Prospective cohort study	Definition: Prevalence of poor sleep quality.<br><br>Instrument(s):	- Sleep quality 54%, (n=124)

	R/E%:NP<br>SO:NP<br>J: National Collegiate Athletic Association (NCAA)<br>S: Soccer		- Sleep quality: PSQI, cut-off (≥5)	
Biggins et al. (2017)<br>[20]	C:<br>A:<br>N:<br>G:<br>R/E%:NP<br>SO:NP<br>J:	Prospective observational study	Definition: Prevalence of poor sleep quality.<br><br>Instrument(s):<br>- Sleep quality: PSQI, cut-off (≥5)	Poor sleep 47.8%, (n=33)
Bilgoe et al (2024)<br>[21]	C: Various<br>A: 25.0± 2.7<br>N: 74<br>G: 0/74<br>R/E%:NP<br>SO:NP<br>J: Current professional football players<br>S: Football	Observational prospective cohort	Definition: Prevalence of symptoms of sport-related psychological distress, anxiety, depression, sleep disturbance, alcohol misuse, substance misuse and disordered eating.<br>Instruments:<br>- sport-related psychological distress APSQ (≥17)<br>- Anxiety: GAD-7 (≥10)<br>- Depression: PHQ-10 (≥10)<br>- Sleep disturbance ASSQ (≥8)<br>- Alcohol misuse: Audit-C : male (≥4), female (≥3)<br>- Substance misuse: CAGE-AID (≥2)<br>- Disordered eating: Beda-Q (≥2)	sport-related psychological distress:64.9% (n=48)<br>- Anxiety: 6.8% (n=5)<br>- Depression: 5.4% (n=3.9)<br>- Sleep disturbance: 24.3% (n=17.9)<br>- Alcohol misuse: 45.9% (n=33.9)<br>- Substance misuse: 1.4% (n=1.03)<br>- Disordered eating: 17.6% (n=13.02)
Bilgoe et al 2025)<br>[74]	C: Netherlands<br>A: former male: 40± 9.3, former female: 35.3± 7.6 ,Current male: 28± 5.4,Current female: 27.8± 6.0 current:45, former: 29<br>N: current:45, former: 29<br>G: current 37/8, former 22/7<br>R/E%:NP<br>SO:NP	Observational cross-sectional	Definition: Prevalence of symptoms of sport-related psychological distress, anxiety, depression, sleep disturbance, alcohol misuse, substance misuse and disordered eating.<br>Instruments:<br>- sport-related psychological distress APSQ (≥17),	Current<br>Sport-related psychological distress: 57.0% (n=29)<br>- Anxiety: 10.2% (n=5)<br>- Depression: 8.7% (n=4)<br>- Sleep disturbance: 18.6% (n=8)<br>- Alcohol misuse: 16.7% (n=8)<br>- Substance misuse: 7.0% (n=3)

	J: Current and former elite kickboxers<br>S: Kickboxing		former:K10<br>- Anxiety: GAD-7 (≥10)<br>- Depression: PHQ-9 (≥10)<br>- Sleep disturbance ASSQ (≥8)<br>- Alcohol misuse: Audit-C : male (≥4), female (≥3)<br>- Substance misuse: CAGE-AID (≥2)<br>- Disordered eating: Beda-Q (≥2), former ESP (≥2)	- Disordered eating: 62.8% (n=27)<br><br>Former<br>Sport-related psychological distress:25.7% (n=9)<br>- Anxiety: 6.1% (n=2)<br>- Depression: 3.1% (n=1)<br>- Sleep disturbance: 36.7% (n=11)<br>- Alcohol misuse: 42.9% (n=15)<br>- Substance misuse: 12.9% (n=4)<br>- Disordered eating: 26.7% (n=8)
Bilgoe et al. (2024)<br>[22]	C: Netherlands<br>A: 26.0 ± 7.5<br>N: 26.0 ± 7.5<br>G: 71/85<br>R/E%:NP<br>SO:NP<br>J: Current elite athletes<br>S: Olympic and paralympic sports	Cross-sectional	Definition: Prevalence of symptoms of sport-related psychological distress, anxiety, depression, sleep disturbance, alcohol misuse, substance misuse and disordered eating.<br>Instruments:<br>- sport-related psychological distress APSQ (≥17)<br>- Anxiety: GAD-7 (≥10)<br>- Depression: PHQ-10 (≥10)<br>- Sleep disturbance ASSQ (≥8)<br>- Alcohol misuse: Audit-C : male (≥4), female (≥3)<br>- Drug misuse: CAGE-AID (≥2)<br>- Disordered eating: Beda-Q (≥2)	sport-related psychological distress:73.4% (n=102)<br>- Anxiety: 13.5% (n=18)<br>- Depression: 17.2% (n=22)<br>- Sleep disturbance: 24.6% (n=30)<br>- Alcohol misuse: 51.7% (n=62)<br>- Drug misuse: 1.7% (n=2)<br>- Disordered eating: 15.7% (n=19)
Brown et al. (2017)<br>[65]	C: France, Ireland and South Africa<br>A: 39 yrs (SD=5) voluntary, 37 yrs (SD= 5) forced<br>N: 173 (voluntary retirement), 120 (forced retirement)<br>G:	Observational prospective cohort	Definition: Prevalence of symptoms of distress, anxiety/depression, sleep disturbance, and adverse alcohol use.<br><br>Instrument(s):	Prevalence voluntary retirement;<br>- Distress 18.9% (31.66)<br>- Anxiety/depression 26.1% (45.15)<br>- Sleep disturbance 23.9% (41.35)<br>- Adverse alcohol use 21.9%

	R/E%:NP<br>SO:NP<br>J: Former elite athletes<br>S: Rugby		- Distress :4DSQ (≥4)<br>- Anxiety/depression: GHQ-12 (≥3)<br>- Sleep disturbance: PROMIS Sleep (≥13)<br>- Adverse alcohol use: AUDIT-C (≥5)	(37.89)<br><br>Prevalence forced retirement;<br>- Distress 32.8% (39.36)<br>- Anxiety/depression 32.7% (39.24)<br>- Sleep disturbance 34.5% (41.4)<br>- Adverse alcohol use 26.3% (31.56)
Dorfling et al. (2024)<br>[23]	C: New Zealand<br>A: majority, 88 (83.8%) aged 20–29 years<br>N: 105<br>G: 105/0<br>R/E%:44 NZ European (41.9%), 21 Māori (20%), 36 Pacifica (34.3%) and 4 other (3.8%)<br>SO:NP<br>J: Current elite athletes<br>S: Rugby	Cross-sectional	Definition: Prevalence of symptoms of depression, anxiety and alcohol misuse.<br><br>Instrument(s):<br>- Depression: PHQ-9 (≥ 10)<br>- Anxiety: GAD-7 (≥ 10)<br>- alcohol misuse: ASSIST (≥ 6)	Depression: 21% (n=22)<br>Anxiety: 17.1% (n=18)<br>alcohol misuse: 51.4% (n=54)
Drew et al (2017)<br>[24]	C: Australia<br>A: Male: 25.8 ±4.1; Female: 24.3 ±3.9<br>N: 132<br>G: 47/85<br>R/E%:NP<br>SO:NP<br>J: Current elite athletes<br>S: Olympic sports (boxing, equestrian, football, gymnastics, hockey, rowing, rugby sevens, sailing, triathlon, and water polo)	Observational cross-sectional	Definition: Prevalence of poor sleep quality, and poor mental health symptoms<br><br>Instrument(s):<br>- Depression: DASS-21 subscale (>4)<br>- Anxiety: DASS-21 subscale (>3)<br>- Stress: DASS-21 subscale (>8)<br>- Sleep quality: PSQI, cut off (≥5)<br>- Sleepiness: ESS, cut-off (>10)	- Depression 17 % (n=22)<br>- Anxiety 19 % (n=24)<br>- Stress 14 % (n=18)<br>- Poor sleep 49 % (n=51)<br>- Sleepiness 22 % (n=23)
Drew et al (2019)<br>[25]	C: Dublin<br>A: Male: 20.32±2.17; Female: 21.57±2.91<br>N: 185	Cross-sectional	Definition: Prevalence of symptoms of depression and Anxiety	Depression: Male 13.7% (n=16), Female 21.5% (n=14)<br>Anxiety: Male 23.9% (n=28), Female 29.2% (n=19)

	G: 117/65<br>R/E%:NP<br>SO:NP<br>J: Current elite student athletes<br>S: Gaelic Football, hurling, basketball, track and field, swimming, golf, triathlon, canoeing, equestrian		Instrument(s):<br>- Depression: DASS-D subscale (≥14)<br>- Anxiety: DASS-A subscale (≥12)	
Du Preez et al. (2017)<br>[26]	C:Australia<br>A: 21.4 ±3.6<br>N: 404<br>G: 404/0<br>R/E%:European 193<br>Pasifika 116<br>Indigenous 43<br>European-Pasifika 17<br>Middle-Eastern 13<br>Other 10 not reported<br>SO:NP<br>J: Current elite athletes<br>S: Rugby	Cross-sectional	Definition: Prevalence of depression, generalized anxiety, and alcohol misuse during pre-season and in-season<br><br>Instrument(s):<br>- Depression: PHQ-9 (≥10)<br>- Anxiety/depression: GAD-7 (≥10)<br>- Alcohol Misuse: AUDIT-C (≥4)	pre-season<br>- Depression 12.6% (n=51)<br>- Anxiety 14.6% (n=59)<br>- Alcohol Misuse 68.6 % (n=251)<br>in season<br>- Depression 10.1% (n=28)<br>- anxiety 10.1% (n=28)<br>- alcohol misuse: 62.8% (n=157)
Escobar-molina et al (2015)<br>[27]	C: Spain<br>A: 21.4 ±3.0<br>N: 68<br>G: 37/31<br>R/E%:NP<br>SO:NP<br>J: Current elite athletes<br>S: Judo	Cross-sectional	Definition: Mean scores of anxiety and eating attitudes<br><br>Instrument(s):<br>- Anxiety: STAI-T (NR)<br>- Eating attitudes: EAT-40 (NR)	Mean Scores;<br>- Anxiety 17.7 (SD=6.9)<br>- Eating attitudes 12.2 (SD=9.1)
Foskett et al (2018)<br>[28]	C:Uk<br>A: 24.0 ± 8.6<br>N: 143<br>G: 81/61*<br>R/E%:NP<br>SO:NP<br>J: Current elite athletes<br>S: Athletics, Basketball, Beach volleyball, Biathlon, Boxing, Cricket, Curling, Cycling,	Cross-sectional	Definition: Prevalence of signs of distress and anxiety/depression<br><br>Instrument(s):<br>- Distress :4DSQ (≥4)<br>- Anxiety/depression: GHQ-12 (≥2)	Distress: 26.8% (n=38)<br>Anxiety/depression: 47.8% (n=66)

	Fencing, Football, Gymnastics, Hockey, Ice hockey, Mixed martial arts, Netball, Orienteering, Rowing, Rugby league, Rugby union, Swimming, Table tennis, Triathlon, Volleyball, Water skiing, Wrestling			
Fröhlich et al. (2021) [29]	C: Switzerland<br>A: 24.0±5.2<br>N: 203<br>G: 111/92<br>R/E%:NP<br>SO:NP<br>J: Current elite athletes<br>S: Athletics, boxing, cycling, equestrian, fencing, floorball, football, handball, judo, orienteering, shooting, swimming, triatlon, alpine skiing, biathlon, bobsleigh, cross-country, skiing, freestyle skiing, ice hockey, nordic combined skeleton, ski jumping, snowboarding, speed skating	Cross-sectional	Definition: Prevalence of depression, trait anxiety, state anxiety and disordered sleep during the Covid-19 period.<br><br>Instrument(s):<br>- Depression: PHQ-9 (≥10)<br>- Anxiety: StAI<br>- Sleep disorders: ISI (>15)	- Depression 7 % (n=13)<br>- Trait anxiety: 27.2 ±11.0 (mean±SD)<br>- State anxiety: 29.2 ±11.4 (mean±SD)<br>- disordered sleep: 4% (n=7)
Gerber et al (2023) [30]	C; Switzerland<br>A: 16.76±1.36<br>N: 135<br>G: 93/42<br>R/E%:NP<br>SO:NP<br>J: Current elite athletes<br>S: Soccer, Handball, Judo, Track and field, Floorball, Swimming, Tennis, Volleyball, Cycling, Ice hockey, karate, trampoline, basketball, dancing.	Longitudinal observational study	Definition: Prevalence of symptoms of depression, general burnout, insomnia<br><br>Instrument(s):<br>- Depression: PHQ-9 (≥10)<br>- General burnout symptoms: SMBM (≥4.40)<br>- Sleep disorders: ISI (>15)	Depression 16.3% (n=22)<br>general burnout symptoms: 13.3% (n=18)<br>Insomnia: 4.4% (n=6)
Giel et al. (2016) [31]	C: Germany<br>A: 16.3±1.1	Observational cross-sectional	Definition: Prevalence of depression/anxiety, and eating	Prevalence (among individuals non-weight dependent sports);

	N: 994<br>G: NR<br>R/E%:NP<br>SO:NP<br>J: Current elite athletes<br>S: Technical sports, endurance sports, aesthetic sports, weight dependent sports, ball games, and power sports		disorder pathology<br><br>Instrument(s):<br>- Depression/Anxiety: PHQ-4 (>5)<br>- Eating Disorders: FKKS (<19), SIAB-S (>1 compensatory behavior), and SCOFF (>1 positive answer)	- Eating Disorder Pathology 27.9% (274)<br>- Anxiety/depression 6.9% (75)
Gouttebarge et al (2017)<br>[32]	C: Netherlands<br>A: 27 ± 7<br>N: 203<br>G: 73/130<br>R/E%:Dutch<br>SO:NP<br>J: Current elite athletes<br>S: Mixed Olympic and national team athletes	Observational cross-sectional	Definition: prevalence of symptoms of distress, anxiety/depression, sleep disturbance, adverse alcohol use and disordered eating<br><br>Instrument(s):<br>- Distress :4DSQ (≥4)<br>- Anxiety/depression: GHQ-12 (≥3)<br>- Sleep disturbance: PROMIS Sleep (≥13)<br>- Adverse alcohol use: AUDIT-C (≥5)<br>- Eating disorders: Eating Disorder Screen - Primary Care (≥2)	prevalence;<br>- Distress 27% (n= 54.81)<br>- Anxiety/depression 45% (n= 91.35)<br>- Sleep disturbance 22% (n=44.66)<br>- Adverse alcohol use 6% (n=12.18)<br>- Eating disorders 32 % (n= 64.96)
Gouttebarge et al. (2015)<br>[75]	C: Australia, Ireland, The Netherlands, New Zealand, Scotland and the USA<br>A: current: 27±5, former: 36±5<br>N: current: 149, former: 104<br>G: current: 149/0, former: 104/0<br>R/E%:NP<br>SO:NP<br>J: Current and former professional football players<br>S; Football (soccer)	Observational cross-sectional	Definition: 6-month prevalence of symptoms of low self-esteem and burnout, 1-month prevalence of distress, anxiety/depression, and point prevalence of smoking, and adverse alcohol use.<br><br>Instrument(s):<br>- Distress :4DSQ (≥4)<br>- Anxiety/depression: GHQ-12 (≥3)<br>- Burnout: Utrecht Burn-Out	Current 1-month prevalence;<br>- Distress 10% (15)<br>- Anxiety/depression 26% (38)<br><br>Current 6-month prevalence;<br>- Burnout 5% (7)<br>- Low self-esteem 3% (5)<br><br>Current point prevalence;<br>- Smoking 7% (10)<br>- Adverse alcohol use 19% (28)<br><br>Former

			Scale (>75% percentile from the reference population)<br>- Adverse alcohol use: AUDIT-C (≥5)	- Distress 18% (n=19)<br>- Anxiety/depression 39% (n=41)<br>- Adverse alcohol use 32% (n=33)
Gouttebarge et al. (2015)<br>[33]	C: Various<br>A: 26.8±4.4<br>N: 607<br>G: 607/0<br>R/E%:NP<br>SO:NP<br>J: Current Professional Football players<br>S: Football (soccer)	Observational cross-sectional	Definition: 4-week prevalence of symptoms of distress, anxiety/depression, sleep disturbance, and adverse alcohol use.<br><br>Instrument(s):<br>- Distress: 4DSQ (≥4)<br>- Anxiety/depression: GHQ-12 (≥3)<br>- sleep disturbance: PROMIS, short form (≥1)<br>- Adverse alcohol use: AUDIT-C (≥5)	Distress 14.8% (n= 81)<br>Anxiety/depression 37.9% (n=187)<br>Sleep disturbance 23.4% (n= 128)<br>Adverse alcohol use 9.4% (n= 50)
Gouttebarge et al. (2016)<br>[66]	C: Belgium, Chile, Finland, France, Japan, Norway, Paraguay, Peru, Spain, Sweden Switzerland<br>A: 35.0 ± 6.4<br>N: 219<br>G: 219/0<br>R/E%:NP<br>SO:NP<br>J: Retired professional footballers<br>S: Football (soccer)	Observational cross-sectional	Definition: Prevalence of symptoms of distress, anxiety/depression, and adverse alcohol use.<br><br>Instrument(s):<br>- Distress :4DSQ (≥4)<br>- Anxiety/depression: GHQ-12 (≥3)<br>- Sleep disturbance: PROMIS Sleep (≥13)<br>- Adverse alcohol use: AUDIT-C (≥5)	Prevalence;<br>- Distress 18.4% (n=38)<br>- Anxiety/depression 35.3% (n=66)<br>- Sleep disturbance 28.2% (n=58)<br>- Adverse alcohol use 24.6% (n=50)
Gouttebarge et al. (2016)<br>[67]	C: France, Ireland and South Africa<br>A: 38±6<br>N: 295<br>G: 295/0<br>R/E%:NP<br>SO:NP	Observational cross-sectional	Definition: Prevalence of symptoms of distress, anxiety/depression, and adverse alcohol use.<br><br>Instrument(s):<br>- Distress :4DSQ (≥4)	Prevalence;<br>- Distress 24.8% (n= 69)<br>- Anxiety/depression 28.4% (n=79)<br>- Sleep disturbance 28.8% (n=70)

	J: Retired Rugby union players<br>S: Rugby		- Anxiety/depression: GHQ-12 (≥3)<br>- Sleep disturbance: PROMIS Sleep (≥13)<br>- Adverse alcohol use: AUDIT-C (≥5)	- Adverse alcohol use 23.8 %(n=65)
Gouttebarge et al. (2016)<br>[34]	C: Ireland<br>A: 25 ± 4<br>N: 204<br>G: 204/0<br>R/E%:NP<br>SO:NP<br>J: Current elite athletes<br>S: Gaelic sports (hurling/football)	Observational cross-sectional	Definition: 4-week prevalence of symptoms of distress, anxiety/depression, sleep disturbance, and adverse alcohol use.<br><br>Instrument(s):<br>- Distress :4DSQ (≥4)<br>- Anxiety/depression: GHQ-12 (≥3)<br>- Sleep disturbance: PROMIS Sleep (≥13)<br>- Adverse alcohol use: AUDIT-C (≥5)	prevalence;<br>- Distress 38.3% (72)<br>- Anxiety/depression 47.8% (87)<br>- Sleep disturbance 33.0% (62)<br>- Adverse alcohol use 23.2% (41)
Gouttebarge et al. (2017)<br>[35]	C: Argentina, Australia, Canada, England, France, Ireland, Italy, New Zealand, Pacific Islands, South Africa, USA, Wales<br>A: 25.0±4.0<br>N: 990<br>G: 941/49<br>R/E%:NP<br>SO:NP<br>J: Current professional rugby player<br>S: Rugby	Cross-sectional	Definition: 4-week prevalence of symptoms of distress, anxiety/depression, sleep disturbance, eating disorders, and adverse alcohol use.<br><br>Instrument(s):<br>- Distress :4DSQ (≥4)<br>- Anxiety/depression: GHQ-12 (≥3)<br>- Sleep disturbance: PROMIS Sleep (≥13)<br>- Adverse alcohol use: AUDIT-C (≥5)<br>- Eating disorders: Eating Disorder Screen - Primary Care (≥2)	Distress 17% (n= 168)<br>Anxiety/depression 30% (n=297)<br>Sleep disturbance 13% (n= 128)<br>Adverse alcohol use 15% (n=148)<br>Eating disorders 23% (n=227)
Gouttebarge et al. (2017)	C: Denmark, Finland, Norway, Switzerland	Observational cross-sectional	Definition: 4-week prevalence of symptoms of distress,	Current: prevalence;<br>- Distress 13.4% (n=18.09)

[76]	A: Current: 26.0±5.0 , Retired: 35.0±8.0<br>N: Current: 135 , Retired: 123<br>G: 135/0, 123/0<br>R/E%:NP<br>SO:NP<br>J: Current and retired professional athletes<br>S: Ice hockey		anxiety/depression, sleep disturbance, adverse alcohol use and disordered eating<br><br>Instrument(s):<br>- Distress :4DSQ (≥4)<br>- Anxiety/depression: GHQ-12 (≥3)<br>- Sleep disturbance: PROMIS Sleep (≥13)<br>- Adverse alcohol use: AUDIT-C (≥5)<br>- Eating disorders: Eating Disorder Screen - Primary Care (≥2)	- Anxiety/depression 24.1% (n=32.5)<br>- Sleep disturbance 15.0% (n=20)<br>- Adverse alcohol use 7.6% (n=10.26)<br>- Eating disorders 17.6 %(n=23.76)<br><br>Retired: prevalence;<br>- Distress 11.7 %(n= 14.3)<br>- Anxiety/depression 19.4% (n=23.8)<br>- Sleep disturbance 16.7 %(n= 20.5)<br>- Adverse alcohol use 28.7 %(n=35.3)<br>- Eating disorders 23.8 %(n=29.2)
Gouttebarge et al (2017)<br>[77]	C: Netherlands<br>A: Current athletes: 27.3 ± 7.1, Former athletes: 50.7±15.1<br>N: Current athletes: 203, Former athletes: 282<br>G: Current athletes: 73/130, Former athletes: 138/144<br>R/E%:NP<br>SO:NP<br>J: Current elite athletes and Former elite athletes<br>S: Mixed Olympic and national team athletes	Observational cross-sectional	Definition: Prevalence of symptoms of distress, anxiety/depression, sleep disturbance, adverse alcohol use, and eating disorder.<br><br>Instrument(s):<br>- Distress :4DSQ (≥4)<br>- Anxiety/depression: GHQ-12 (≥3)<br>- Sleep disturbance: PROMIS Sleep (≥13)<br>- Adverse alcohol use: AUDIT-C (≥5)<br>- Eating disorders: Eating Disorder Screen - Primary Care (≥2)	Current athletes: Distress 26.6% (n=51)<br>Anxiety/depression 44.7% (n=84)<br>Sleep disturbance 22.3% (n=42)<br>Adverse alcohol use 6.4% (n=12)<br>Eating disorder 32.1% (n=60)<br>Former athletes: Distress 17.5% (n=50)<br>Anxiety/depression 29.4% (n=83)<br>Sleep disturbance 21.8% (n=61)<br>Adverse alcohol use 23.2% (n=64)<br>Eating disorder 27.4% (n=76)
Gouttebarge et al. (2018)<br>[36]	C: Argentina, Australia, Canada, England, France, Ireland, Italy, New Zealand, Pacific Islands	Observational prospective cohort	Definition: prevalence of symptoms of distress, anxiety/depression, sleep	Prevalence;<br>- Distress 20% (n=119)<br>- Anxiety/depression 32% (n=

	(including Fiji, Samoa, Tonga), South Africa, USA, Wales<br>A: 26.0±4.0<br>N: 595<br>G: 595/0<br>R/E%:NP<br>SO:NP<br>J: Current professional athletes<br>S: Rugby		disturbance, eating disorders, and adverse alcohol use.<br><br>Instrument(s):<br>- Distress :4DSQ (≥4)<br>- Anxiety/depression: GHQ-12 (≥3)<br>- Sleep disturbance: PROMIS Sleep (≥13)<br>- Adverse alcohol use: AUDIT-C (≥5)<br>- Eating disorders: Eating Disorder Screen - Primary Care (≥2)	190.4)<br>- Sleep disturbance 12% (n=71.4)<br>- Adverse alcohol use 15% (n=89.25)<br>- disordered eating 21% (n=124.95)
Gouttebarge et al. (2022)<br>[37]	C: Netherlands<br>A: Non covid group<br>Male: 24.8±4.5, Female: 23.1±3.9<br>Covid group<br>Male: 26.0±5.0, Female 22.8±3.9<br>N: 1602<br>G: 1134/468<br>R/E%:NP<br>SO:NP<br>J: Current professional footballers<br>S: Football	Comperative cross-sectional study	Definition: Two week prevalence of anxiety and depressive symptoms in a non-Covid group and a Covid-19 group<br><br>Instrument(s):<br>- Anxiety: GAD-7 (≥10)<br>- Depression; PHQ-9 (≥10)	Non covid group<br>Depression: Male 5.7% (n=10), Female 10.6% (n=14)<br>Anxiety; Male 4% (n=7), Female 8.4% (n=11)<br>Covid group<br>Depression: Male 12.8% (n=145), Female 21.6% (n=101)<br>Anxiety; Male 15.6% (n=177), Female 18.1% (n=84)
Gulliver et al. (2015)<br>[38]	C: Australia<br>A: 24.9 ± 6.0<br>N: 224<br>G: 106/118<br>R/E%:NP<br>SO:NP<br>J: Current elite athletes<br>S: Cricket, football (soccer), hockey, netball, rowing, sailing, water polo, softball, athletics/track and field, cycling	Observational cross-sectional	Definition: Prevalence of general psychological distress and common mental disorders.<br><br>Instrument(s):<br>- Distress: K-10 (≥22)<br>- Anxiety: GAD-7 (≥11)<br>- Depression: CES-D (≥16)<br>- Social anxiety: SPIN (≥19)<br>- Panic disorder: PDSS-SR	Prevalence;<br>- Distress:16.5 % (n=37)<br>- Anxiety: 7.1 (n=16)<br>- Depression:27.2% (n=61)<br>- Social anxiety:14.7% (n=33)<br>- Panic disorder: 4.5 % (n=10)<br>- Eating disorders: 22.8 % (n=51)

	(track and road), skiing (alpine and aerial), volleyball, golf, powerlifting, archery, beach volleyball, canoe slalom, gymnastics, swimming, triathlon, basketball, kayak, orienteering, skeleton		(≥19)<br>- Eating disorders: SCOFF (≥2)	
Hakansson et al. (2018)<br>[39]	C: Sweden<br>A: 23.7 ± 3.2<br>N: 352<br>G: 141/211<br>R/E%:NP<br>SO:NP<br>J: Current elite athletes<br>S: Athletics; martial arts; cross-country skiing; handball; canoeing; alpine skiing; gymnastics	Observational cross-sectional	Definition: Prevalence of symptoms of alcohol misuse<br><br>Instrument(s):<br>- Adverse alcohol use: AUDIT-C (≥5 male; ≥4 female)	Prevalence;<br>- Adverse alcohol use 26% (n=91)
Hart et al. (2013)<br>[68]	C: USA<br>A: 61.8 yrs (57.8–65.7)<br>N: 34<br>G: 34/0<br>R/E%: Caucasian (n=23), African-american (n=11),<br>SO:NP<br>J: Former professional players<br>S: American Football	Observational cross-sectional	Definition: Diagnosis of depression<br><br>Instrument(s):<br>- Depression: BDI-II (NR)	Prevalence;<br>- Depression: 24% (8)
Henderson (2023)<br>[40]	C: Australia<br>A: 22.06± 3.05<br>N: 369<br>G: 337/32<br>R/E%: Australian 86.2%, Aboriginal or Torres Strait Islander 10.8%, other 3%<br>SO:NP<br>J: Current elite athletes<br>S: Football	Cross-sectional	Definition: Prevalence of symptoms of depression and anxiety.<br><br>Instrument(s):<br>- Depression: PHQ-9 (≥10)<br>- Anxiety: GAD-7 (≥10)	Prevalence:<br>- Anxiety: 1.12% (n=4)<br>- Depression: 13.76% (n=49)
Identig (2024)<br>[41]	C: Sweden<br>A: 25.8 ± 8.4	Cross-sectional	Definition: Prevalence of symptoms of depression,	Prevalence:<br>- Depression 27.6% (n=13)

	N: 47<br>G: 24/23<br>R/E%:Swedish<br>SO:NP<br>J: Current elite athletes<br>S: Rock-climbing		anxiety, stress and sleep disturbance.<br><br>Instrument(s):<br>- Depression: DASS-21 (≥7)<br>- Anxiety: DASS-21 (≥5)<br>- Stress: DASS-21 (≥10)<br>- Sleep quality: PSQI (≥5)	- Anxiety 23.5% (n=11)<br>- Stress 55.3 % (n=26)<br>- sleep disturbance 49 % (n=23)
Junge et al. (2016)<br>[42]	C: Zwitserland<br>A: 22.3 ± 4.47<br>N: 471<br>G: 289/182<br>R/E%:NP<br>SO:NP<br>J: Current elite athletes<br>S: Football (soccer)	Observational cross-sectional	Definition: Prevalence of symptoms of depression and anxiety.<br><br>Instrument(s):<br>- Depression: CES-D (≥16)<br>- Anxiety: GAD-7 (≥10)	Prevalence;<br>- Depression, 10.6% (n=46)<br>- Anxiety, 1.4% (n=6)
Junge et al. (2023)<br>[43]	C: Germany<br>A: 20.0 ± 4.7<br>N: 187<br>G: 105/82<br>R/E%: German<br>SO:NP<br>J: Current elite athletes<br>S: Field hockey	Observational cross-sectional	Definition: Prevalence of symptoms of depression and anxiety .<br>Instruments:<br>- Anxiety: GAD-7 (≥10)<br>- Depression: CES-D (≥16)	Prevalence:<br>- Anxiety: 1.2% (n=1)<br>- Depression: Female 18.3% (n=15), male 4.8% (n=5)
Kerr et al. (2012)<br>[69]	C: USA<br>A: NP<br>N: 2536 (2001), 1316 (2010)<br>G: 2536/0<br>R/E%:NP<br>SO:NP<br>J: Former professional athletes<br>S: American Football	Observational prospective cohort	Definition: Prevalence of clinical diagnosis of depression<br>Instrument(s):<br>- Depression: GHS questions	2010 prevalence;<br>- Depression 10.2% (n= 106)
Kuetell et al. (2021)<br>[44]	C: Denmark<br>A: : 34.0 ± 4.9<br>N: 612<br>G: 354/258<br>R/E%: Danish<br>SO:NP<br>J: Current elite athletes	Observational cross-sectional	Definition: Prevalence of symptoms of anxiety and depression.<br>Instruments:<br>- Anxiety: GAD-7 (≥10)<br>- Depression: CES-D (≥16)	Prevalence:<br>- Anxiety: 13.9% (n=85)<br>- Depression: 21.1% (n=129)

	S: Badminton, cycling, swimming, tennis, basketball, football, handball, ice-hockey, volleybal.			
Kilic et al. (2017) [78]	C: Denmark<br>A: Football<br>Current: 25.8 ± 4.9<br>Retired: 34.0 ± 4.9<br><br>Handball<br>Current: 25.3 ± 4.5<br>Retired: 35.0 ± 5.6<br>N: football<br>Current players (N = 348)<br>Retired players (N= 345)<br><br>Handball<br>Current players (N = 232)<br>Retired players (N= 230)<br>G: Current :285/63 football, 118/114 handball<br>Former: 79/21 football, 100/0 handball<br>R/E%:Danish<br>SO:NP<br>J: Current and former elite athletes<br>S: Football, handball	Observational cross-sectional	Definition: Prevalence of symptoms of distress, anxiety/depression, sleep disturbance, and adverse alcohol use.<br><br>Instrument(s):<br>- Distress :4DSQ (≥4)<br>- Anxiety/depression: GHQ-12 (≥3)<br>- Sleep disturbance: PROMIS Sleep (≥13)<br>- Adverse alcohol use: AUDIT-C (≥5)	Current:<br>Prevalence football;<br>- Distress 14.7 % (n=51.15)<br>- Anxiety/depression 18.1 % (n=62.9)<br>- Sleep disturbance 15.8 % (n= 54.9)<br>- Adverse alcohol use 2.9 % (n=10.09)<br>Prevalence handball;<br>- Distress 19.8 % (n=45.9)<br>- Anxiety/depression 26.3 % (n=61.0)<br>- Sleep disturbance 22.0 % (n=51.04)<br>- Adverse alcohol use 2.6 % (n=6.03)<br><br>Former:<br>Prevalence football;<br>- Distress 8.7% (n=30.01)<br>- Anxiety/depression 18.7% (n= 64.51)<br>- Sleep disturbance 11.0 %(n=73.95)<br>- Adverse alcohol use 8.4% (n= 28.98)<br>Prevalence handball;<br>- Distress 16.3% (n=37.49)<br>- Anxiety/depression 15.8% (n= 36.34)<br>- Sleep disturbance 12.2% (n=28.06)<br>- Adverse alcohol use 7.0% (n=16.1)

Kilic et al. (2021)<br>[79]	C: Australia<br>A: Current: 25.8 ± 4.9<br>N: Current: 281, Former: male 81<br>G: Current: 149/132, former 81/0<br>R/E%:NP<br>SO:NP<br>J: Current and former professional footballers<br>S: Football (soccer)	Observational comparitive cross-sectional	Definition: Prevalence of symptoms of sport-related psychological distress, global psychological distress, anxiety, depression, sleep disturbance, alcohol misuse, gambling problem and disordered eating.<br>Instruments:<br>- sport-related psychological distress APSQ (≥17)<br>- Global psychological distress K-10 (≥21)<br>- Anxiety: GAD-7 (≥10)<br>- Depression: PHQ-10 (≥10)<br>- Sleep disturbance ASSQ (≥8)<br>- Alcohol misuse: Audit-C : male (≥4), female (≥3)<br>- Problem gambling; NODS-clip (≥1)<br>- Disordered eating: Beda-Q (≥4)<br>- Substance misuse: CAGE-AID (≥2)	Prevalence:<br>Current professional footballers:<br>Sport-related psychological distress: male 52% (n= 77), Female 62.9% (n=83)<br>Global psychological distress: male 9.5% (n=14), female 18.9% (n= 25)<br>Anxiety: male 4.7% (n= 7), female 8.3% (n= 11)<br>Depression: male 6.8% (n= 10), female 10.6% (n=14)<br>Sleep disturbance: male 12.2% (n=18), female 32.6% (n= 43)<br>Alcohol misuse: male 50.7% (n= 76), female 43.8% (n= 58)<br>Substance misuse: male: 2.0% (n=3), female 1.5% (n=2)<br>Problem gambling: male 23.6% (n= 35), female 2.3% (n=3)<br>Disordered eating: male: 35.1%, (n= 52), female 43.8% (n=58)<br>Former professional footballers:<br>Global psychological distress: 26.3% (n= 21)<br>Anxiety: 11.3% (n=9)<br>Depression: 12.5 % (n= 10)<br>Sleep disturbance: 32.5 % (n=26)<br>Alcohol misuse: 68.8% (n= 56)<br>Substance misuse: 10.6% (n= 9)<br>Problem gambling: 32.5% (n=26)<br>Disordered eating: 40.0% (n=32)
Kruger et al. (2025)<br>[45]	C: South Africa<br>A: 20.45 ± 1.93<br>N: 200	Cross-sectional	Definition: Prevalence of symptoms of stress, anxiety, depression, sleep disturbance,	stress: 21% (n=42)<br>Anxiety: 45% (n=90)<br>Depression: 24% (n=48)

	G: 72/128<br>R/E%: Asian 0.5%, black 25.5%, coloured 9.5%, white 64.5%<br>SO:NP<br>J: Current student athletes<br>S: Field hockey, netball, athletics (track and field), badminton, basketball, water polo, gymnastics, judo, swimming, rowing and fencing.		alcohol misuse, and disordered eating.<br>Instruments:<br>- stress: DASS-21 (≥19)<br>- Anxiety: DASS-21 (≥10)<br>- Depression: DASS-21 (≥14)<br>- Sleep disturbance ASSQ (≥8)<br>- Alcohol misuse: Audit-C : male (≥3), female (≥2)<br>- Disordered eating: Beda-Q (≥4)	Sleep disturbance 35% (n=70)<br>Alcohol misuse: 29% (n=58)<br>Disordered eating: 75% (n=150)
Lima et al. (2022) [46]	C: Turkey<br>A: np<br>N: 579<br>G: 311/268<br>R/E%:turkish<br>SO:NP<br>J: Current elite athletes<br>S: Soccer, volleyball, handball, badminton, basketball, field hockey, martial arts, athletics, swimmers, tennis, fencing	Cross-sectional	Definition: prevalence of symptoms of distress, anxiety and depression.<br>Instruments:<br>- sport-related psychological distress APSQ (≥17)<br>- Anxiety: GAD-7 (≥10)<br>- Depression: PHQ-10 (≥10)	Prevalence:<br>- sport-related psychological distress: 71.7% (n=415)<br>- Anxiety: 21.1% (n=122)<br>- Depression: 30.1% (n=174)
Lima et al. (2024) [47]	C: Turkey<br>A: 16.42 ± 0.49<br>N: 301<br>G: 205/96<br>R/E%:turkish<br>SO:NP<br>J: Current elite athletes<br>S: 22 different sport disciplines (e.g., football, volleyball, wrestling)	Cross-sectional	Definition: prevalence of symptoms of distress, anxiety and depression.<br>Instruments:<br>- sport-related psychological distress APSQ (≥17)<br>- Anxiety: GAD-7 (≥10)<br>- Depression: PHQ-10 (≥10)	Prevalence:<br>- sport-related psychological distress: 61.5% (n=185)<br>- Anxiety: 29.9% (n=90)<br>- Depression: 33.2% (n=117)
Moore et al. (2025) [48]	C: USA<br>A: 19.87 ± 1.47<br>N: 62<br>G: 19/42, other:1<br>R/E%:77.4% (n = 48) Caucasian/White, 8.1% (n = 5) African American/Black, 8.1%	Cross-sectional	Definition: Prevalence of symptoms of anxiety, depression, anxiety and alcohol misuse<br><br>Instrument(s):<br>- Anxiety: GAD-7 (≥ 10)	Anxiety: 25.8% (n=16)<br>depression: 14.5% (n=9)<br>alcohol misuse: 21.2% (n=14)

	(n = 5) Multiple ethnic/other), and 6.5% (n = 4) Hispanic<br>SO:NP<br>J: Current student athletes<br>S: Varsity level organized sports		- depression: PHQ-9 (≥ 10)<br>- alcohol misuse: audit (≥ 8)	
Mountjoy et al. (2023)<br>[49]	C: Canada<br>A: 25.3 ± 4.5<br>N: T0: 543, T1:336, T2: 133<br>G: T0:294/248, T1:146/190, T2: 44/89<br>R/E%:NP<br>SO:NP<br>J: Current elite athletes<br>S: Baseball, basketball, field hockey, football, hockey, lacrosse, rugby, soccer, wrestling, curling, cross country, figure skating, golf, track and field, rowing, nordic skiing, swimming.	Cross-sectional with 3 repeated measurements	Definition: Prevalence of symptoms of sport-related psychological distress, anxiety, depression, sleep disturbance, alcohol misuse, substance misuse and disordered eating.<br>Instruments:<br>- sport-related psychological distress APSQ (≥17)<br>- Anxiety: GAD-7 (≥10)<br>- Depression: PHQ-10 (≥10)<br>- Sleep disturbance ASSQ (≥8)<br>- Alcohol misuse: Audit-C : male (≥4), female (≥3)<br>- Substance misuse: CAGE-AID (≥2)<br>- Disordered eating: Beda-Q (≥4)	Prevalence at T0:<br>- Distress: male 19.4% (n= 57) , female 30.2% (n= 74)<br>- Anxiety: male 24.6% (n= 72) , female 33.3% (n= 82)<br>- Depression: male 24.6% (n=72) , female 26.7% (n= 66)<br>- Sleep disturbance: male 38.6% (n=113) , female 40.0% (n= 99)<br>- Alcohol misuse: male 50.9% (n=149) , female 57.3% (n=142)<br>- Substance misuse: male 17.5% (n= 51) , female 4.0% (n= 9)<br>- Eating disorders: male 80.7% (n= 237) , female 84.0% (n=208)<br><br>Prevalence at T1:<br>- Distress: male 33.9% (n= 49) , female 45.2% (n=85)<br>- Anxiety: male 8.2% (n=11) , female 19.0% (n= 36)<br>- Depression: male 8.2% (n= 11) , female 25.0% (n=47)<br>- Sleep disturbance: male 26.5% (n= 38) , female 34.5% (n=65)<br>- Alcohol misuse: male 57.1% (n= 83) , female 53.6% (n= 101)

				- Substance misuse: male 14.3% (n= 20) , female 4.8% (n= 9)<br>- Eating disorders: male 71.4% (n= 104) , female 84.5% (n= 160)<br><br>Prevalence at T2:<br>- Distress: male 27.0% (n= 11) , female 36.7% (n= 32)<br>- Anxiety: male 27.3% (n= 12), female 28.1% (n=25)<br>- Depression: male 9.1% (n=4) , female 31.3% (n= 27)<br>- Sleep disturbance: male 0 (n= 0) , female 31.3% (n= 27)<br>- Alcohol misuse: male 63.6% (n= 27) , female 43.8% (n= 38)<br>- Substance misuse: male 9.1% (n= 4) , female 3.1% (n= 2)<br>- Eating disorders: male 63.6% (n= 27) , female 75.0% (n= 66)
Nixdorf et al. (2013)<br>[50]	C: Germany<br>A: 35.0 ± 5.6<br>N: 134<br>G: 78/56<br>R/E%:NP<br>SO:NP<br>J: Current elite athletes<br>S: Badminton, ice running, golf, athletics, modern penthalon, cycling, wrestling, swimming, snowboarding, triathlon, beach volleyball, icw hockey, soccer, handball, hockey, rugby, volleyball, canoeing	Cross-sectional	Definition: Prevalence of symptoms of depression.<br>Instruments:<br>- Depression: CES-D (>22)	Depression: 19% (n= 25)

Orzali et al.<br>(2025)<br>[51]	C: Sweden<br>A: 13.3 ± 1.1<br>N: 351<br>G: 0/351<br>R/E%:NP<br>SO:NP<br>J: Current elite athletes<br>S: soccer	Cross-sectional	Definition: Prevalence of symptoms of distress.<br><br>Instrument(s):<br>- Distress: GHQ-12 (≥ 3)	Distress: 22% (n=76)
Perry et al.<br>(2022)<br>[52]	C: England<br>A: 25.1± 4.5<br>N: 115<br>G: 0/115<br>R/E%:NP<br>SO:NP<br>J: Current elite athletes<br>S: Football	Cross-sectional	Definition: Prevalence of symptoms of depression, anxiety and disordered eating.<br><br>Instrument(s):<br>- Depression: PHQ-9 (≥10)<br>- Anxiety: GAD-7 (≥10)<br>- Disordered eating: BEDA-Q (≥4)	Prevalence:<br>- Depression: 11.2% (n=11)<br>- Anxiety: 11% (n=11)<br>- Disordered eating: 36% (n=36)
Pillay et al<br>(2024)<br>[53]	C: Netherlands<br>A: 26.5 ± 1.7<br>N: 101<br>G: 101/0<br>R/E%:NP<br>SO:NP<br>J: Current professional football players<br>S: Football	Observational prospective cohort study	Definition: Prevalence of symptoms of sport-related psychological distress, anxiety, depression, sleep disturbance, alcohol misuse, substance misuse and disordered eating.<br>Instruments:<br>- sport-related psychological distress APSQ (≥17)<br>- Anxiety: GAD-7 (≥10)<br>- Depression: PHQ-10 (≥10)<br>- Sleep disturbance ASSQ (≥8)<br>- Alcohol misuse: Audit-C : male (≥4), female (≥3)<br>- Drug misuse: CAGE-AID (≥2)<br>- Disordered eating: Beda-Q (≥2)	sport-related psychological distress:52.5% (n=53)<br>- Anxiety: 7.9% (n=8)<br>- Depression: 7.9% (n=8)<br>- Sleep disturbance: 16.8% (n=17)<br>- Alcohol misuse: 47.5% (n=48)<br>- Substance misuse: 5.9% (n=6)<br>- Disordered eating: 12.9% (n=13)
Poucher et al.<br>(2022)<br>[54]	C: Canada<br>A: 26.0± 4.4<br>N: 186<br>G: 37/113<br>R/E%:NP	Cross-sectional	Definition: Prevalence of symptoms of depression, anxiety and disordered eating at 4 time points.	Prevalence at T1:<br>- Depression: 17.2% (n=32)<br>- Anxiety: 6.5% (n=12)<br>- Disordered eating: 3.2% (n=6)

	SO:NP<br>J: Current elite athletes<br>S: NP		Instrument(s):<br>- Depression: CESD-R (≥ 16)<br>- Anxiety: GAD-7 (≥10)<br>- Disordered eating: EAT (≥ 20)	Prevalence at T2:<br>- Depression: 13.4% (n=19)<br>- Anxiety: 5.6% (=8)<br>- Disordered eating: 0.7% (n=1)<br><br>Prevalence at T3:<br>- Depression: 17.1% (n=21)<br>- Anxiety: 5.7% (n=7)<br>- Disordered eating: 0.8% (n=1)<br><br>Prevalence at T4:<br>- Depression: 10.6% (n=13)<br>- Anxiety: 4.6% (n=5)<br>- Disordered eating: 1.9% (n=2)
Purcell et al. (2022) [55]	C: Australia<br>A: 24.6± 6.9<br>N: 749<br>G: 344/405<br>R/E%:NP<br>SO: Heterosexual 92.5%, Bisexual 2.9%, same sex attracted 1.9%<br>J: Current elite athletes<br>S: Team and individual sports	Cross-sectional	Definition: Prevalence of symptoms of distress and alcohol misuse.<br><br>Instrument(s):<br>- Distress: K-10 (≥ 22)<br>- Alcohol misuse: Audit-c (≥5)	Prevalence:<br>- Distress: 17.7% (n=132)<br>- Alcohol misuse: 15.3% (n=114)
Saju et al. (2025) [56]	C: India<br>A: male 19.74± 1.73, female 19.30± 1.56<br>N: 262<br>G: 183/79<br>R/E%:NP<br>SO:NP<br>J: Current collegiate athletes<br>S: Athletics, badminton, basketball, combat sports, cricket, football, hockey, kabaddi, kho-kho, rowing, shooting, silambam (an Indian martial art), swimming, throwball, and volleyball	Cross-sectional	Definition: Prevalence of symptoms of depression and anxiety .<br>Instruments:<br>- Anxiety: GAD-7 (≥9)<br>- Depression: PHQ-9 (≥10)	Prevalence:<br>- Anxiety: male 54.0% (n=98) , female 46.0% (n=36)<br>- Depression: male 70.0% (n=128) , female 30.0% (n= 23)

Sanders et al. (2017) [70]	C: UK<br>A: 46.8 ± 15.7 years<br>N: 307<br>G: 307/0<br>R/E%:NP<br>SO:NP<br>J: Retired professional athletes<br>S: Football (soccer)	Observational cross-sectional	Definition: Prevalence of possible clinically relevant depressive symptoms<br>Instrument(s):<br>- Depression: SDHS (<10)	7-day prevalence;<br>- Depression 15.6 % (48)
Schaal et al. (2011) [57]	C: France<br>A: 18.5 ± 4.9<br>N: 2067<br>G: 1338/728<br>R/E%:French<br>SO:NP<br>J: Current elite athletes<br>S: Mixed Olympic sports	Observational cross-sectional and retrospective	Definition: prevalence of symptoms of anxiety, depression, sleep disturbance, eating disorders, and substance abuse.<br><br>Instrument(s):<br>- Clinical diagnosis (physician or psychologist) according to DSM-IV criteria	Prevalence lifetime;<br>- Generalised anxiety 8 (NR)<br>- depression 2.6 (NR)<br>- eating disorders 6.2 (NR)<br>- Sleep problems (>15 days) 26.6 (NR)<br>- Psychosis 0.4 (NR)<br>- Substance abuse / dependence 4.1 (NR)<br><br>Prevalence current;<br>- Generalised anxiety 6 (NR)<br>- depression 0.7 (NR)<br>- eating disorders 4.3 (NR)<br>- Sleep problems (>15 days) 21.5 (NR)<br>- Psychosis 0.2 (NR)<br>- Substance abuse / dependence 0 (NR)
Schuring et al. (2017) [71]	C: South Africa<br>A: 36.7± 6.3<br>N: 624<br>G: NP<br>R/E%:NP<br>SO:NP<br>J: Former elite athletes<br>S: Cricket, football, rugby, ice hockey, gaelic sports	Cross-sectional	Definition: Prevalence of symptoms of distress, anxiety/depression, sleep disturbance and adverse alcohol use.<br><br>Instrument(s):<br>- Distress :4DSQ (≥4)<br>- Anxiety/depression: GHQ-12 (≥4)<br>- Sleep disturbance: PROMIS Sleep (≥2)	Distress 22.1% (n=133)<br>Anxiety/depression 27.1% (n=163)<br>Sleep disturbance 26.9% (n=161)<br>Adverse alcohol use 26.2% (n= 157)

			- Adverse alcohol use: AUDIT-C (≥5)	
Schuring et al. (2017)<br>[80]	C: South Africa<br>A: former: 36 ± 6.0, Current: 27 ± 5.0<br>N: Current:78 (baseline),53 (6 month follow up), Former:38 (baseline), 23 (6-month follow-up)<br>G: Current 68/10, Former 38/0<br>R/E%:NP<br>SO:NP<br>J: Current and former elite athletes<br>S: Cricket	Observational prospective cohort	Definition: 4-week prevalence, and 6-month incidence of symptoms of distress, anxiety/depression, sleep disturbance, and adverse alcohol use.<br><br>Instrument(s):<br>- Distress :4DSQ (≥4)<br>- Anxiety/depression: GHQ-12 (≥3)<br>- Sleep disturbance: PROMIS Sleep (≥13)<br>- Adverse alcohol use: AUDIT-C (≥5)	Prevalence;<br>- Distress 38.4 (28.0–49.8)<br>- Anxiety/depression 37.0 (26.8–49.1)<br>- Sleep disturbance 38.4 (28.0–49.8)<br>- Adverse alcohol use 26.0 (17.3–37.2)<br><br>6-month incidence;<br>- Distress 9.4 (2.5–25.0)<br>- Anxiety/depression 15.6 (6.4–32.2)<br>- Sleep disturbance 15.2 (6.2–31.4)<br>- Adverse alcohol use 15.6 (6.4–32.2)
Schwenk et al. (2007)<br>[72]	C: USA<br>A: 53.4 ± 14.5<br>N: 1617<br>G: 1617/0<br>R/E%:NP<br>SO:NP<br>J: Former professional athletes<br>S: American Football	Observational cross-sectional	Definition: Prevalence of symptoms of depression<br><br>Instrument(s):<br>Depression: PHQ-9 (≥10)	Prevalence;<br>- Depression 14.7% (n=237)
Tan et al. (2016)<br>[58]	C; England, Scotland, Wales<br>A: 16.70 ± 2.46 years<br>N: 51<br>G: 16/35<br>R/E%:NP<br>SO:NP<br>J: Current elite athletes<br>S: Gymnastics	Observational cross-sectional	Definition: Identify symptom patterns of eating disorder and depression.<br><br>Instrument(s):<br>Depression: BDI-II (10-18, ≥19)<br>Eating problem: EAT-26 (20)	Prevalence current;<br><br>Depression, moderate 6% (3)<br>Eating problem 19 %(9)
Thompson et al. (2024)<br>[59]	C: USA<br>A: NP<br>N: 1216<br>G: 796/419	Cross-sectional	Definition: Prevalence of symptoms of depression,anxiety, sleep disturbance, eating disorder,	Depression: 3.3% (N= 40)<br>- Anxiety: 4.4% (n= 53)<br>- Sleep disturbance: 16.7% (n= 145)

	R/E%:NP<br>SO:NP<br>J: Current collegiate athletes<br>S: Golf, tennis, basketball, track and field, volleyball,field hockey, soccer, baseball, football		and substance abuse<br><br>Instrument(s):<br>- Depression: PHQ-9 (≥ 10)<br>- Anxiety: GAD-7 (≥ 10)<br>- Sleep disturbance: PROMIS Sleep (≥ 21)<br>- Substance abuse: CAGE-AID (≥ 3)<br>- Eating disorders: SCOFF, ESP (≥ 3)	- Substance abuse: 2.4% (n=21)<br>- Eating disorders: 6.0% (n= 72)
Uriegas et al (2023)<br>[60]	C: USA<br>A: 19.8± 1.4<br>N: 1885<br>G: 573/1312<br>R/E%:NP<br>SO:NP<br>J: Current collegiate athletes<br>S: Endurance, aesthetic, power, ball and team, technical	Cross-sectional	Definition: Prevalence of symptoms of disordered eating<br><br>Instrument(s):<br>- Disordered eating: EAT-26 (≥ 20)	Disordered eating: 22.7% (n= 428)
Valster et al. (2022)<br>[61]	C: Chicago<br>A: NP<br>N: T1:533, T2: 535<br>G: T1: 351/182, T2:344/191<br>R/E%:NP<br>SO:NP<br>J: Current collegiate athletes<br>S: Cross-country, golf, tennis, track and field, wrestling, baseball, basketball, football, soccer, softball and volleyball	Non-experimental trend study design	Definition: Prevalence of symptoms of depression and anxiety at 2 time points. (1 year and 2 years)<br><br>Instrument(s):<br>- Depression: HANDS (≥9)<br>- Anxiety: BAI (≥21)	Prevalence at T1:<br>- Depression: 9.2% (n=49)<br>- Anxiety: 1.9% (n=10)<br><br>Prevalence at T2:<br>- Depression: 9.5% (n=51)<br>- Anxiety: 2.0% (n=10)
Willer et al. (2018)<br>[73]	C: USA<br>A: Contact sport athlete group (mean age 56.7 years), non contact sports group (mean age 55.4 years).<br>N: 21 (contact sports), 21 (non contact sports group)<br>G: NP	Case-control	Definition: clinical diagnosis of depression and anxiety .<br>Instruments:<br>- Anxiety: BAI(≥10)<br>- Depression: BDI-I (≥14)	Depression: 23.8% (n=5)<br>Anxiety: 33.3% (n=7)

	R/E%:NP<br>SO:NP<br>J: Former professional athletes<br>S: Football, Hockey			
Wilson et al.<br>(2022)<br>[62]	C: Northeastern USA<br>A: 19.5 ± 1.13<br>N: 218<br>G: 140/78<br>R/E%:NP<br>SO:NP<br>J: Current collegiate athletes<br>S: Football, soccer, lacrosse, baseball, volleyball, field hockey, softball, basketball, wrestling	Cross-sectional	Definition: Prevalence of symptoms of depression, stress and anxiety.<br><br>Instrument(s):<br>- Depression: PHQ-9 (≥ 10)<br>- Stress: DASS-21 (≥10)<br>- Anxiety: DASS-21 (≥ 6)	Depression: 23.2% (n=33)<br>- Stress: 30.8% (n=67)<br>- Anxiety: 40.9% (n=89)
Wolanin et al.<br>(2015)<br>[63]	C: USA<br>A: NP<br>N: 465<br>G: 199/263<br>R/E%: Caucasian 88.8%, African-american 6.2%, Asian 2.8%, other 2.2%<br>SO:NP<br>J: Current collegiate athletes<br>S: Track and field, chearleading, crew, tennis, basketball, soccer, baseball/softball, field hockey, lacrosse	Cross-sectional	Definition: Prevalence of symptoms of depression.<br><br>Instrument(s):<br>- Depression: CES-D(≥ 16)	Depression: Male 22.3% (n=44), Female 35.6% (n=93)
Yang et al.<br>(2007)<br>[64]	C: USA<br>A: 20 ± 1.3<br>N: 257<br>G: 167/90<br>R/E%: white 89.9%, other 10.1%<br>SO:NP<br>J: Current collegiate athletes<br>S: Football, baseball, wrestling, spirit squad, rowing, basketball, gymnastics, golf, field hockey,	Prospective cohort study	Definition: Prevalence of symptoms of depression.<br><br>Instrument(s):<br>- Depression: CES-D(≥ 16)	Depression: 30.3% (n=79)

	tennis, cross country, track and field			

C, country; A, age; N, number of participants; G, gender; R/E, race/ethnicity; SO, sexual orientation; J, type athletes; S, sport

Supplementary material S6: Prevalence of mental health symptoms among entourage members: data extraction from included studies

Authors (year)	Study population	Study design	Outcome measurements (symptoms, instruments, threshold)	Prevalence %
Åkesdotter et al. (2022) [14]	C: Sweden<br>A: 42.4±8.8<br>N: 29<br>G: 20/9<br>R/E%:NP<br>SO:NP<br>J: Coach<br>S: Team sports, for example, soccer and ice hockey, and individual sports, such as swimming and gymnastics<br>Level S: High-performance	Descriptive observational design	Definition: Prevalence of psychiatric disorders, namely affective disorders, anxiety disorders, eating disorders, disorders due to psychoactive substance use, alcohol-related disorders<br><br>Instrument(s): diagnostic interviews based on the following:<br>- Any affective disorder: ICD-10:F30-F39<br>- Any Anxiety disorder : ICD-10: F40-F43<br>- Any Eating disorder: ICD-10: F50<br>- Any Disorder due to psychoactive substance use:<br>- Alcohol-related disorders: F10,Z721	-Any affective disorder: total 52% (n=29), Female 78% (n=9), male 40% (n=20)<br>- Any Anxiety disorder : total 93% (n=29), Female 100% (n=9), male 90% (n=20)<br>- Any Eating disorder: total n<5 (n=29), Female n<5 (n=9), male n<5(n=20)<br>- Any disorder due to psychoactive substance use: total 17% (n=29), Female n<5 (n=9), male n<5 (n=20)<br>- Alcohol-related disorders: total n<5 (n=29), Female n<5 (n=9), male n<5 (n=20)
Bilgoe et al. (2024) [22]	C: Netherlands<br>A: 45.3±10.4<br>N: 95<br>G: 74/21<br>R/E%:NP<br>SO:NP<br>J: Coach<br>S: Olympic and Paralympic sports<br>Level S: Elite	Cross-sectional	Definition: Prevalence of symptoms of distress, anxiety, depression, sleep disturbance, alcohol misuse, substance misuse and disordered eating.<br>Instruments:<br>- Distress K-10 (≥21)<br>- Anxiety: GAD-7 (≥10)<br>- Depression: PHQ-10 (≥10)<br>- Sleep disturbance ASSQ (≥8)<br>- Alcohol misuse: Audit-C : male (≥4), female (≥3)	- Distress: 40.7% (n= 35)<br>- Anxiety: 4.7% (n= 4)<br>- Depression: 4.7% (n=4)<br>- Sleep disturbance: 23.5% (n=19)<br>- Alcohol misuse: 53.1% (n= 43)<br>- Drug misuse: 1.3% (n=1)<br>- Disordered eating: 0% (n=0)

			- Drug misuse: CAGE-AID (≥2)<br>- Disordered eating: ESP (≥2)	
Eken et al. (2023) [6]	C: Tokyo, Beijing<br>A: majority 32%:35-44 years<br>N: 256<br>G: 145/111<br>R/E%:NP<br>SO:NP<br>J: Medicine 55%<br>Physiotherapy 28%<br>Nutrition 1%<br>Nursing 2%<br>Strength and conditioning 2%<br>Other 7%<br>S: Olympic and Paralympic sports<br>Level S: Olympic	Cross-sectional study	Definition: Prevalence of symptoms of anxiety and depression,<br>Instruments:<br>- Anxiety: GAD-7 (≥10)<br>- Depression: PHQ-10 (≥10)	Prevalence:<br>- Anxiety: 8% (n=21)<br>- Depression: 12% (n=30)
Gorczynski et al. (2019) [81]	C: UK<br>A: 27.87±10.58<br>N: 103<br>G: Men 65 (63.1%)/ Female 37 (35.9%)/ another 1 (1.0%)<br>R/E%:White 68.9% (n=71), Asian 9.7% (n=10), Black 9.7% (n=10), Arab 1% (n=1.0), Other 10.7% (n=11)<br>SO: Heterosexual 82.5% (n=85), Bisexual 6.8% (n=7), Gay and lesbian 10.7%(n=11)<br>J: Coach<br>S: Rugby, Football, Cricket, Netball, Cheerleading/dance, Swimming, Basketball, Hockey, Racket sports (tennis, badminton, squash), Athletics, Cycling, Boxing, Gymnastics, Rowing, Running, Diving , Karate, Lacrosse<br>Level S: Coaching level:	Cross-sectional	Definition: Prevalence of symptoms of distress.<br><br>Instrument(s):<br>- Distress: K-10 (≥20)	Prevalence:<br>- Distress: 49.5% (n=51)

	Beginner 30 (29.1%),Amateur 66 (64.1%), Professional/elite 7 (6.8%)			
Keagelaars et al. (2021)<br>[82]	C: Netherland and Belgium<br>A: 43.03±10.51<br>N: 119<br>G: 97/22<br>R/E%: flemish (n=40) and dutch (n=79)<br>SO:NP<br>J: Coach<br>S: Individual and team sports<br>Level S: Elite	Cross-sectional	Definition: Prevalence of symptoms of depression/anxiety, Distress, sleep disturbance, adverse alcohol use.<br>Instruments:<br>- Depression/anxiety: GHQ-12 (≥3)<br>- Distress: 4DSQ (≥4)<br>- Sleep disturbance: promis (≥13)<br>- Adverse alcohol use: Audit-c (≥5)	Prevalence:<br>- Depression/ anxiety 39.5% (n=47)<br>- Distress 19.3% (n=23)<br>- Sleep disturbance 25.2% (n=30)<br>- Adverse alcohol use 19.3% (n=23)
Kim et al. (2020)<br>[83]	C: New Zealand<br>A: Majority aged under 50 (71%)<br>N: 69<br>G: 57/15<br>R/E%:NP<br>SO:NP<br>S: Individual and team sports<br>Level S: Elite	Cross-sectional	Definition: Prevalence of symptoms of depression<br>Instruments:<br>- Depression: CESD-R (≥16)	Prevalence:<br>- Depression: 14.1% (n=10)
Pilkington et al. (2022)<br>[4]	C: Australia<br>A: HPPS:40.0±9.56<br>Coach: 46.4±8.78<br>N:HPPS: 174, Coach:78<br>G: HPPS:72/98, other:4 ;<br>Coach: 59/19<br>R/E%: HPPS: Australian 83.9%;<br>Coach: Australian 77.8%<br>SO:HPPS heterosexual 96.1%;<br>Coach: Heterosexual 95.1%<br>J: Coach, High-performance directors, physiotherapists, nutritionists, medical doctors, sports psychologists.	Cross-sectional	Definition: Prevalence of symptoms of depression, Distress, sleep disturbance and alcohol misuse.<br>Instruments:<br>- Depression: GHQ-28 (≥5)<br>- Distress: K-10 (≥20)<br>- Sleep disturbance: ASSQ (≥8)<br>- Adverse alcohol use: Audit-c (Male ≥5, Female ≥ 4)	HPPS:<br>Prevalence: Depression 40.1% (n=69)<br>Distress 15.5% (n=27)<br>Sleep disturbance 15.1% (n=26)<br>Alcohol misuse 39.0% (n=67)<br>Coach: Prevalence: Depression 43.6% (n=34)<br>Distress 10.3% (n=8)<br>Sleep disturbance 23.4% (n=18)<br>Alcohol misuse 48.1% (n=37)

	S: Individual and team sports<br>Level S: elite			

C, country; A, age; N, number of participants; G, gender; R/E, race/ethnicity; SO, sexual orientation; J, type job; S, sport
